# Comparing DNA enrichment of proliferating cells following administration of different stable isotopes of heavy water

**DOI:** 10.1038/s41598-017-04404-2

**Published:** 2017-06-22

**Authors:** Don E. Farthing, Nataliya P. Buxbaum, Philip J. Lucas, Natella Maglakelidze, Brittany Oliver, Jiun Wang, Kevin Hu, Ehydel Castro, Catherine V. Bare, Ronald E. Gress

**Affiliations:** 1 National Institutes of Health (NIH), National Cancer Institute (NCI), Experimental Transplantation and Immunology (ETIB), 10 Center Drive, Bethesda, MD 20892 United States; 2 OCRT&ME, 10 Center Drive, Bethesda, MD 20814 United States

## Abstract

Deuterated water (^2^H_2_O) is a label commonly used for safe quantitative measurement of deuterium enrichment into DNA of proliferating cells. More recently, it has been used for labeling proteins and other biomolecules. Our *in vitro - in vivo* research reports important stable isotopic labeling enrichment differences into the DNA nucleosides and their isotopologues (e.g. deoxyadenosine (dA) M + 1, dA M + 2, dA M + 3), as well as tumor cell proliferation effects for various forms of commercially available stable heavy water (^2^H_2_O, H_2_^18^O, and ^2^H_2_^18^O). Using an *in vitro* mouse thymus tumor cell line, we determined that H_2_^18^O provides superior DNA labeling enrichment quantitation, as measured by GC-positive chemical ionization (PCI)-MS/MS. In addition, at higher but physiologically relevant doses, both ^2^H_2_^18^O and ^2^H_2_O down modulated mouse thymus tumor cell proliferation, whereas H_2_^18^O water had no observable effects on cell proliferation. The *in vivo* labeling studies, where normal mouse bone marrow cells (i.e. high turnover) were evaluated post labeling, demonstrated DNA enrichments concordant with measurements from the *in vitro* studies. Our research also reports a headspace-GC-NCI-MS method, which rapidly and quantitatively measures stable heavy water levels in total body water.

## Introduction

Deuterium oxide (^2^H_2_O or D_2_O) has been shown to be a safe and stable form of heavy water used for cell kinetics studies, as it constitutively incorporates into the DNA nucleosides of proliferating cells^1–15^. R. Busch *et al*. have postulated that the *de novo* nucleoside synthesis pathway metabolically incorporates deuterium into the C-H bonds of the deoxyribose moiety of the DNA nucleosides^2^. In addition, labeling with D_2_O has recently been used for studies evaluating other biomolecules (e.g. proteins, peptides, metabolites, lipids)^16–26^. Other forms of stable heavy water (e.g. H_2_^18^O, ^2^H_2_^18^O (D_2_^18^O, doubly labeled)) have also been used for research involving cell kinetics, metabolism, and biomolecule labeling, despite a high cost that may limit wider applicability^19, 21, 22, 27, 28^. Since other labels used in cell proliferation studies, such as bromodeoxyuridine (BrdU) and [^3^H]-thymidine, are not safe to use in clinical studies, and given the expanding applicability of stable heavy water for translational research, we evaluated several commercially available forms of stable heavy water (i.e. D_2_O, H_2_^18^O, D_2_^18^O) and characterized their isotopic enrichments into the T cell DNA base deoxyadenosine (dA, purine nucleoside). The goal of this research was to determine which form of stable heavy water would be best for our translational research studying T cell kinetics, T cell imaging, and D_2_O labeling of other biomolecules. For this report, we use the term cell kinetics to represent studies on T cell proliferation, which can be quantitatively measured by enrichment of deuterium into the DNA nucleosides during T cell division.

Previous T cell kinetics research from our group focused on using D_2_O in a pre-clinical mouse model of graft-versus-host disease (GVHD), with GC-PCI-MS/MS quantitation of the deuterium enrichments into the DNA base deoxyadenosine (dA M0) and its dA M + 1 isotopologue (i.e. molecules that differ in isotopic composition, leading to different molecular weights)^10^. Other researchers have used D_2_O (long-term labeling) or D_2_-glucose (short-term labeling), and measured some form of an isotopologue ratio (e.g. (dA M + 1/ (dA M0 + dA M + 1))) or dA M + 2 for cell kinetics computations^2–6, 8^. However, in using the dA M0 to dA M + 1 isotopologue ratio, we found the accuracy and precision of the quantitation a significant challenge as the MS/MS measurement of the deuterium dA M + 1 enrichment is made above an existing naturally occurring background for dA M + 1. The natural isotopic background of the dA M + 1 moiety is mainly due to stable isotopes of Carbon-13 (1.1%), Nitrogen-15 (0.4%), Oxygen-17 (0.04%) and Deuterium (0.01%) atoms. The natural isotopic background of the dA M + 2 moiety is significantly lower, with contributions mainly from the stable isotope of Oxygen-18 (0.2%) and trace amounts from Carbon-13 (0.006%). Therefore, we hypothesized that using a form of stable heavy water that would lead to DNA isotopic enrichments in the dA M + 2 or dA M + 3 isotopologue would be advantageous for MS/MS quantitation of dA and its isotopologues (i.e. dA M + 2 or dA M + 3). For experiments, we used high turnover cells (e.g. mouse thymus tumor cells), which were labeled *in vitro* with stable heavy water, and normal mouse bone marrow cells, also rapidly dividing cells, extracted from mice that underwent *in vivo* labeling to characterize the different forms of stable heavy water isotopic enrichments into the DNA base deoxyadenosine (dA M0) and its isotopologues.

In both pre-clinical and clinical studies, it is important to know the level of stable heavy water in the matrix (e.g. *in vitro* cell media, mouse drinking water, mouse, and human total body water (urine)), as it can affect isotopic enrichments into DNA as well as potentially alter cellular metabolism^29^. Other investigators using stable heavy water for their research have determined the level in total body water (TBW)^27, 28, 30–39^ using infrared absorption or by utilizing test methods for plasma and urine, which involve using a metal catalyst (e.g. uranium), high temperatures (e.g. 600 °C), lengthy overnight incubations, costly solvents (e.g. ^13^C_3_-Acetone) and MS measurements of the deuterium moiety, which exchanges from the stable heavy water to a flammable gas (e.g. acetylene, hydrogen)^27, 33^. To avoid these tedious and somewhat hazardous techniques, we developed a simple headspace-GC-negative chemical ionization (NCI)-MS method that measures stable heavy water levels in TBW using only 25 µL of urine, saliva, blood, or cell media. The test method is based on a rapid gas phase isotopic exchange of the hydrogen:deuterium (H:D) and ^16^O:^18^O moieties between the stable heavy water sample at a basic pH (~13–14) and acetone solvent, with quantitative measurements using headspace-GC-NCI-MS in full scan mode.

## Results

### JMR4 mouse thymus tumor cell line (*in vitro*), growth curve, and DNA processing

**Figure 1:**
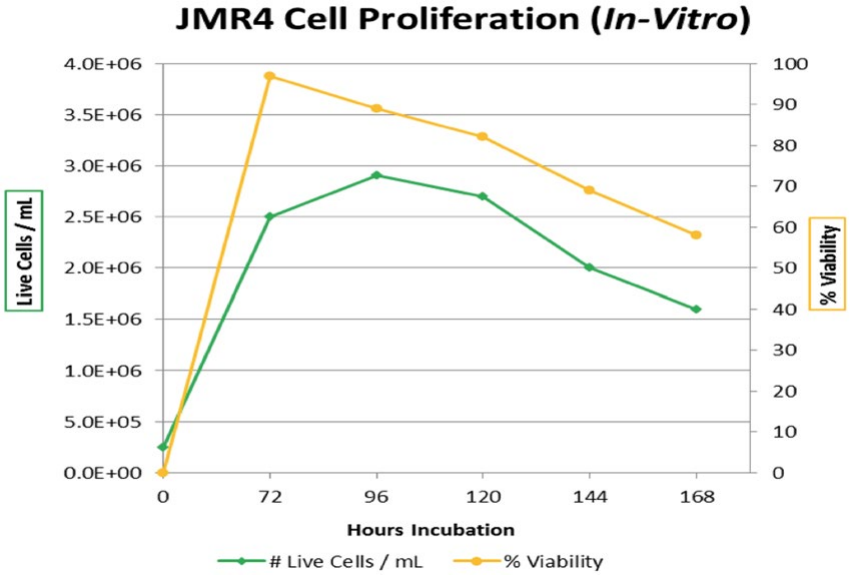
Time course evaluation for JMR4 mouse thymus tumor cell proliferation and cell viability (%). The mouse thymus tumor cells were incubated in cell media (without replacement) for 7 days at 10% CO_2_, > 95% relative humidity and 37 °C. Small aliquots (50 µL) were taken for each time point measurement, with a maximum cell count/mL and viability (%) obtained after 3–5 days. The turnover time for the JMR4 mouse thymus tumor cells in the growth phase and under the stated conditions was approximately 24 hrs. Cell counting and viability (%) using trypan blue staining were measured using the Cellometer™ Auto T4 Cell Counter.

To rapidly obtain information on stable heavy water isotopic enrichments into the DNA of proliferating cells, we used an in-house developed mouse thymus tumor cell line (i.e. JMR4) for all *in vitro* evaluations. These mouse thymus tumor cells, in suspension, were robust and rapidly proliferated in cell media, reaching maximum concentration (live cells/mL) and cell viability (%) after 3–5 days of incubation (Fig. 1). After collecting tumor cells for mass spectrometry (MS) analysis, they were washed several times using PBS (1×, Ca^2+^ and Mg^2+^ free) and centrifugation (200 × g, 3 min, 20 °C), which removed ~99% of the cell media that causes matrix interferences in the DNA hydrolysis procedure. The tumor cells were suspended in a small volume (~50–100 µL) of PBS and rapidly lysed using a novel and recently marketed Episonic™ pulsed sonoporation technology. As seen in Supplementary Information, Figs 1 and 2, the histograms and visual images of pre- and post-pulsed sonoporation of the mouse tumor cells show complete lysis after ~5 min. Subsequently, the DNA was enzymatically hydrolyzed to its nucleoside bases and purified using solid phase extraction, prior to on-line derivatization (methylation) and quantitative GC-PCI-MS/MS analysis.

### D_2_O, H_2_^18^O and D_2_^18^O enrichments into mouse thymus tumor cell DNA nucleosides (*in vitro*)

**Figure 2:**
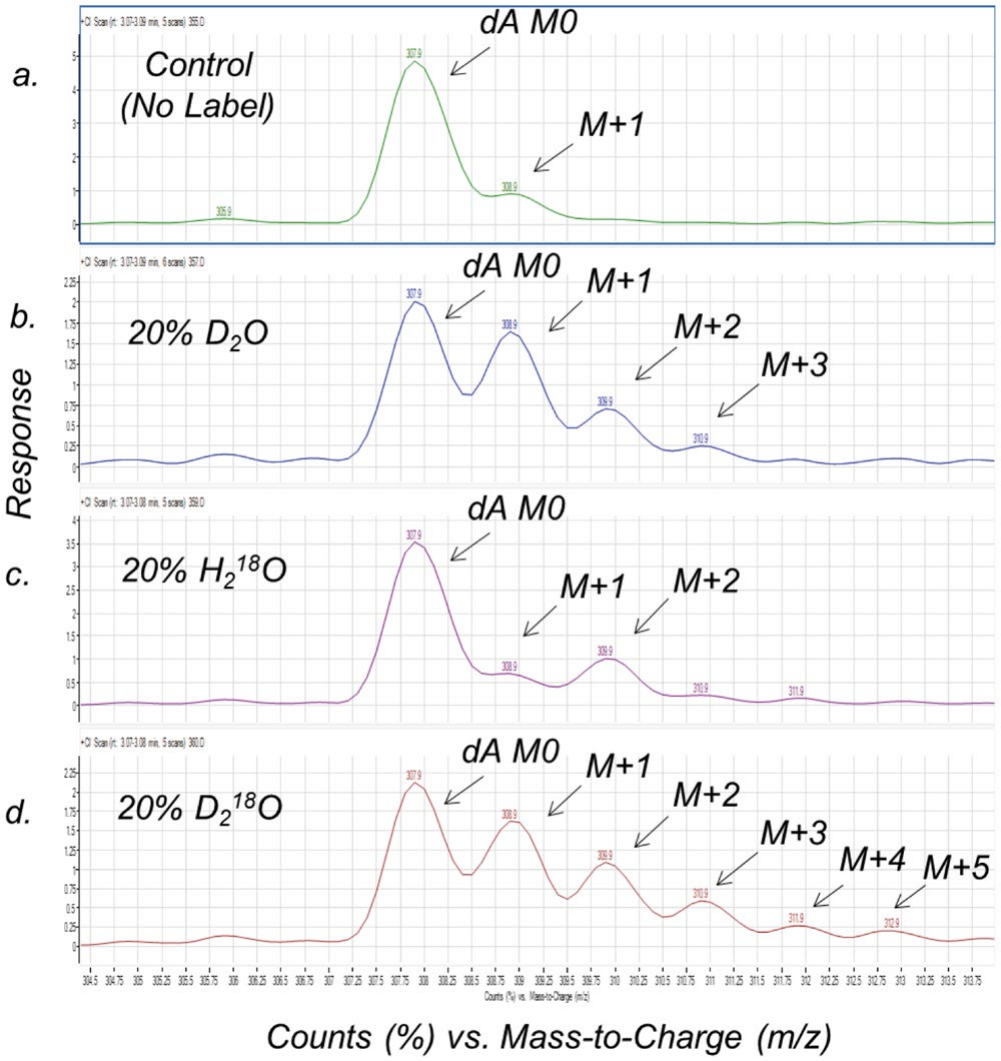
Mass spectrometry isotopic signatures (normalized to dA M0) for heavy water enrichment into mouse thymus tumor cell DNA base deoxyadenosine (dA) after 3–5 days of incubation. (**a**) Mouse thymus tumor cells incubated in cell media demonstrating the natural isotopic abundances of the MethElute™ derivative of dA, with molecular weights of dA M0 (308 m/z) and dA M + 1 (309 m/z). (**b**) Isotopic enrichments (M + 1, M + 2, M + 3) of the tumor cells incubated in cell media fortified with 20% D_2_O (v/v). (**c**) Isotopic enrichment (M + 2) of the tumor cells incubated in cell media fortified with 20% H_2_^18^O (v/v). (**d**) Isotopic enrichments (M + 1, M + 2, M + 3, M + 4, M + 5) of the tumor cells incubated in cell media fortified with 20% D_2_^18^O (v/v).

Using our published analytical method for studying T cell kinetics by measuring D_2_O isotopic enrichments into the DNA base deoxyadenosine (dA)^10^, we initially compared how the different forms of stable heavy water (i.e. D_2_O, H_2_^18^O and D_2_^18^O) enrich into the dA isotopologues (i.e. dA M + 1, dA M + 2, dA M + 3, dA M + 4, dA M + 5). Specifically, we were targeting dA isotopologue enrichments other than dA M + 1, due to it having the highest contribution from natural isotopic background. As seen in Fig. 2, the normalized MS profiles of the dA isotopologues showed vastly different isotopic enrichments from the different forms of stable heavy water into the dA isotopologues. The deuterium enrichments incorporate into all the dA isotopologues (i.e. dA M + 1, dA M + 2, dA M + 3, dA M + 4, dA M + 5), whereas the ^18^O enrichment was detected in the dA M + 2 and dA M + 4 isotopologues. Therefore, as compared to the control sample (Fig. 2a **)**, the use of stable heavy water (H_2_^18^O) would have an optimal MS quantitation target at the dA M + 2 isotopologue (Fig. 2c), which should lead to more accurate and precise quantitation of cell proliferation kinetics.

**Figure 3:**
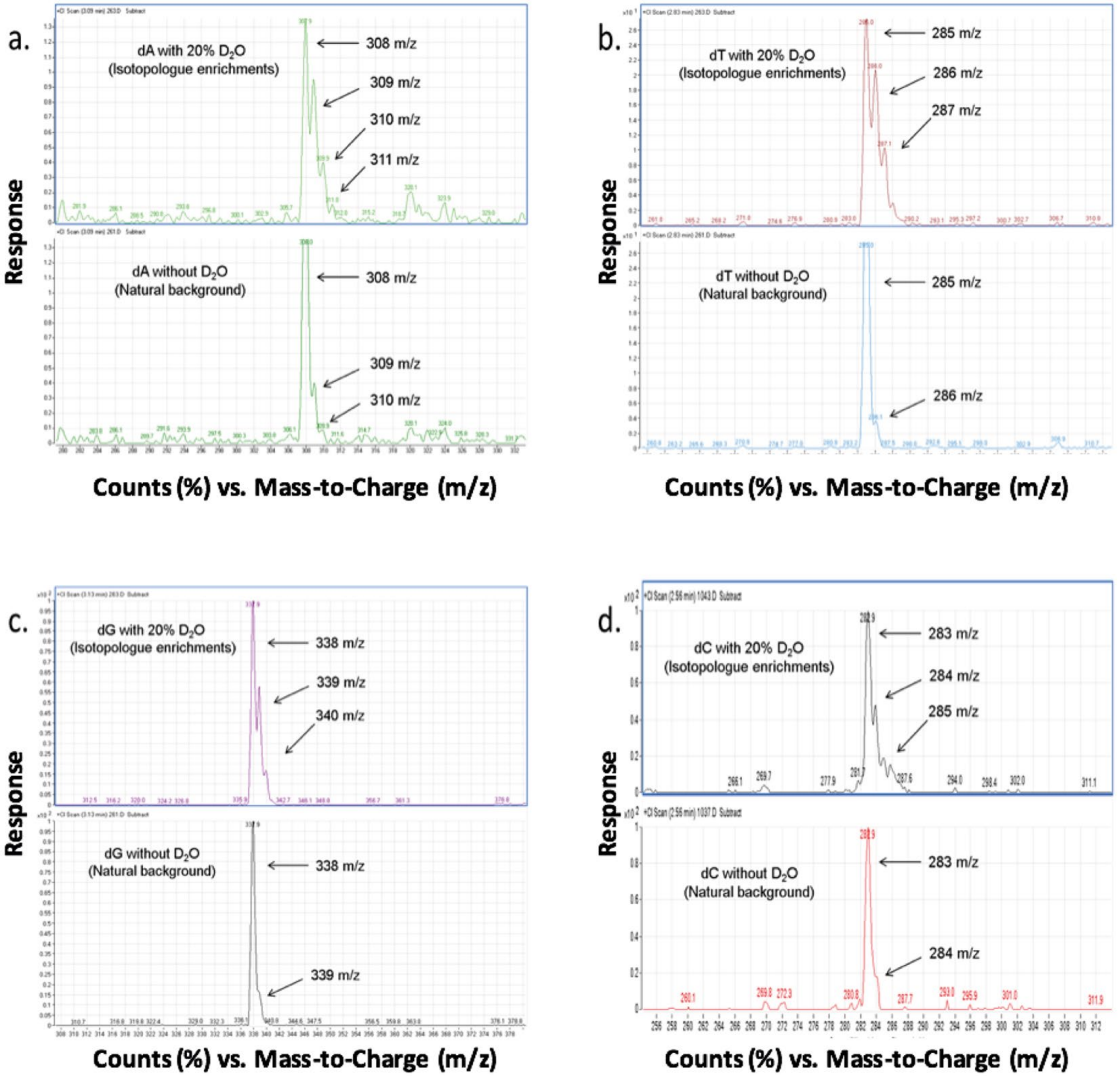
Mass spectrometry isotopic signatures for D_2_O enrichments into the mouse thymus tumor cell DNA nucleosides after 3–5 days of incubation. The mouse thymus tumor cells were incubated in cell media (with and without 20% D_2_O, (v/v)). The natural isotopic background and the deuterium enrichments into the isotopologues (e.g. M + 1, M + 2) are shown for methylated derivatives of (**a**) deoxyadenosine (dA), MW 308 Da, (**b**) deoxythymidine (dT), MW 285 Da, (**c**) deoxyguanosine (dG), MW 338 Da, and (**d**) deoxycytidine (dC), MW 283 Da.

It is important to emphasize that the D_2_^18^O (20%, v/v) generated the largest isotopic enrichments into the dA moiety (e.g. dA M + 1 to dA M + 5), presumably from the enrichments of both the deuterium (D) and oxygen (^18^O) into the deoxyribose and perhaps the adenine moieties of dA (Fig. 2d). As the DNA nucleosides (dA & dG, purines) and (dT & dC, pyrimidines) have similar chemical structures, we decided to evaluate the enrichment of deuterium into the four DNA nucleoside bases from mouse thymus tumor cells dosed with and without D_2_O (20%, v/v). As seen in Fig. 3a–d, each of the DNA nucleosides had large deuterium enrichments into the M + 1 isotopologues (e.g. dG M + 1, dA M + 1, dC M + 1, dT M + 1 etc.), which agrees with stable isotopic enrichments of deuterium occurring via the *de novo* synthesis pathway of each DNA nucleoside.

### Robustness of D_2_O and H_2_^18^O enrichments into the dA isotopologues of mouse thymus tumor cells (*in vitro*)

To evaluate the robustness of D_2_O and H_2_^18^O isotopic enrichments into the dA isotopologues, we used mouse thymus tumor cells and varied the dosing levels (e.g. 1, 5, and 20%, v/v) as well as the number of cells extracted (e.g. 75,000, 150,000, and 300,000) for MS analysis. As demonstrated in Supplementary Information, Fig. 3a and b, the deuterium and H_2_^18^O isotopic enrichments into the dA M + 1 and dA M + 2 isotopologues were very consistent using our *in vitro* experimental conditions and analytical methodology.

### Comparing D_2_O and H_2_^18^O isotopic enrichments into the dA isotopologues of mouse thymus tumor cells (*in vitro*) and normal mouse bone marrow cells (*in vivo*)

For comparing *in vitro* and *in vivo* stable heavy water isotopic enrichments into the dA isotopologues, we dosed D_2_O and H_2_^18^O (~5%, v/v) into cultured mouse thymus tumor cells (*in vitro*) and into BALB/cAnNCr (H-2^d^) mice (*in vivo*). The cultured mouse tumor cells were dosed and incubated as previously described, and the BALB/cAnNCr (H-2^d^) mice were used as approved by the NCI Animal Care and Use Committee for stable heavy water experiments and collection of rapidly dividing bone marrow cells. As demonstrated using these rapidly proliferating cells, evaluation of the different forms of stable heavy water (e.g. D_2_O, H_2_^18^O) can be performed using *in vitro* or *in vivo* experiments (n = 3 for each subset), as they provided comparable isotopic enrichment profiles for the dA isotopologues (i.e. dA M + 1, dA M + 2, dA M + 3, dA M + 4) (Supplementary Information, Fig. 3c and d). Unfortunately, a study of the doubly labeled (D_2_^18^O) water could not be evaluated *in vivo*, due to the large quantities that would be necessary for dosing (bolus and maintenance dose) and the associated high cost of its procurement.

### Time course cell proliferation profiles for mouse thymus tumor cells incubated in D_2_O, H_2_^18^O, and D_2_^18^O water (*in vitro*)

Using mouse thymus tumor cells, we constructed a time course profile (5 days) for cell proliferation (live cells/mL) from acute exposure to the different forms and dosing levels of stable heavy water. For these evaluations, we chose 0% (control), 5% D_2_O (v/v), 20% levels of D_2_O, H_2_^18^O or D_2_^18^O (v/v), and 40% D_2_O (v/v). Samples were taken daily for cell counting and measurement of cell viability (%) using the Cellometer^®^ Auto T4 Cell Counter. As depicted in Supplementary Information, Fig. 4, the mouse thymus tumor cell proliferation rates decreased significantly after 3 days of incubation in cultures containing 20% and 40% D_2_O or 20% D_2_^18^O water, with no observed cell proliferation effect in cultures containing 20% H_2_^18^O. The decrease in the mouse thymus tumor cell proliferation rate when using 20% and 40% D_2_O or 20% D_2_^18^O was an unexpected finding, as other investigators have reported minimal side effects for D_2_O at dosing levels up to 20% (v/v). Further research on the cell proliferation effect at the higher levels (≥20%) of D_2_O and D_2_^18^O is necessary to better understand the biological effects from these higher, yet relevant levels of D_2_O and D_2_^18^O.

### D_2_O enrichment resulting from labeling and de-labeling of mouse thymus tumor cell dA isotopologues (*in vitro*)

**Figure 4:**
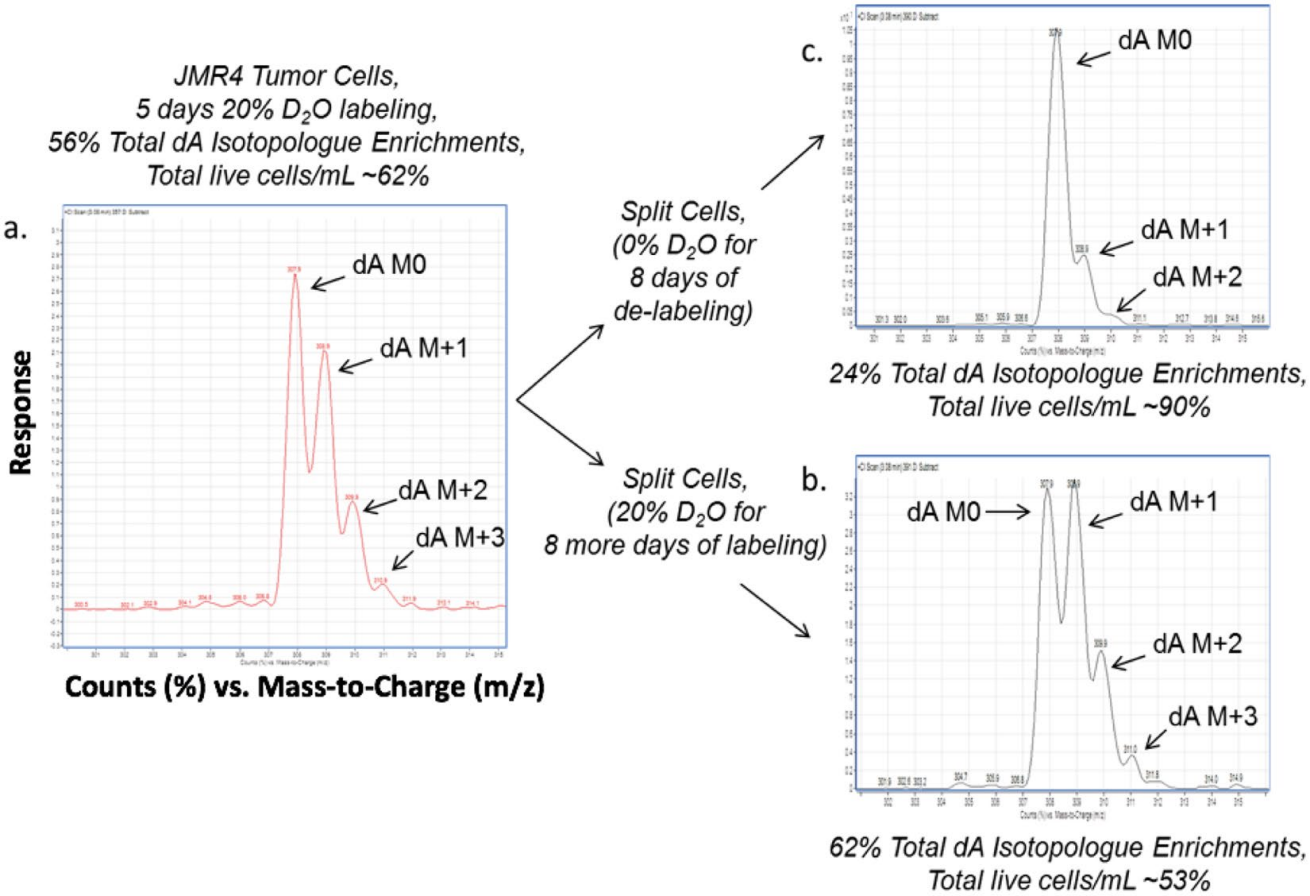
Mouse thymus tumor cells incubated using 20% D_2_O for dA enrichment study. Mouse thymus tumor cells were incubated in cell media using incubator conditions of 10% CO_2_, > 95% relative humidity and 37 °C. The tumor cells were dosed using 20% D_2_O (v/v) and incubated for 5 days; afterwards the cells were split into fresh media (with and without 20% D_2_O) for an additional 8-day incubation. (**a**) Using a 20% D_2_O dosing, the tumor cell DNA base deoxyadenosine (dA) demonstrated significant deuterium enrichment into the dA isotopologues (~56% total dA enrichment), which negatively affected cell proliferation (~62% cell viability). (**b**) With a longer incubation period (13 days total), measurement of the cell counts and % cell viability using 20% D_2_O revealed an increase in total dA isotopologue enrichments (~62%) and further decrease in cell proliferation (~53% cell viability). (**c**) The tumor cells split into fresh media without D_2_O proliferated like control (0% D_2_O) mouse thymus tumor cells (total dA isotopologue level (~24%) and cell viability (~90%)).

A study was performed to determine whether deuterium isotopic enrichments from D_2_O into the dA isotopologues were reversible. Using mouse tumor cells dosed at 20% D_2_O (v/v) for 5 days of incubation, the deuterium enrichments into the dA isotopologues were measured using GC-PCI-MS in full scan mode (Fig. 4). Deuterium enrichments were detected in the dA M + 1 through dA M + 3 isotopologues (Fig. 4a). The mouse tumor cells were then split into fresh cell media with and without 20% D_2_O for an additional 8 days of incubation, and deuterium enrichments into the dA isotopologues were subsequently measured using GC-PCI-MS in full scan mode.

The tumor cells incubated with 20% D_2_O in cell media had increased deuterium enrichment levels into the dA isotopologues (Fig. 4b), indicating an additional accumulation of deuterium enrichments. However, tumor cells incubated without 20% D_2_O in cell media had typical dA isotopologue levels and a cell viability (%) that resembled mouse thymus tumor cells without the stable heavy water label (Fig. 4c). This observation suggests that the deuterium enrichments from D_2_O into the dA isotopologues may be reversible, which is an interesting finding that will initiate future investigative research. During this study, we observed negative effects of D_2_O (20%, v/v) on mouse thymus tumor cell proliferation, as indicated by a lower total cell viability (live cells/mL) as deuterium enrichments increased into the dA isotopologues.

### Heavy water dose-dA isotopic enrichments into mouse thymus tumor cell dA isotopologues (*in vitro*)

**Figure 5:**
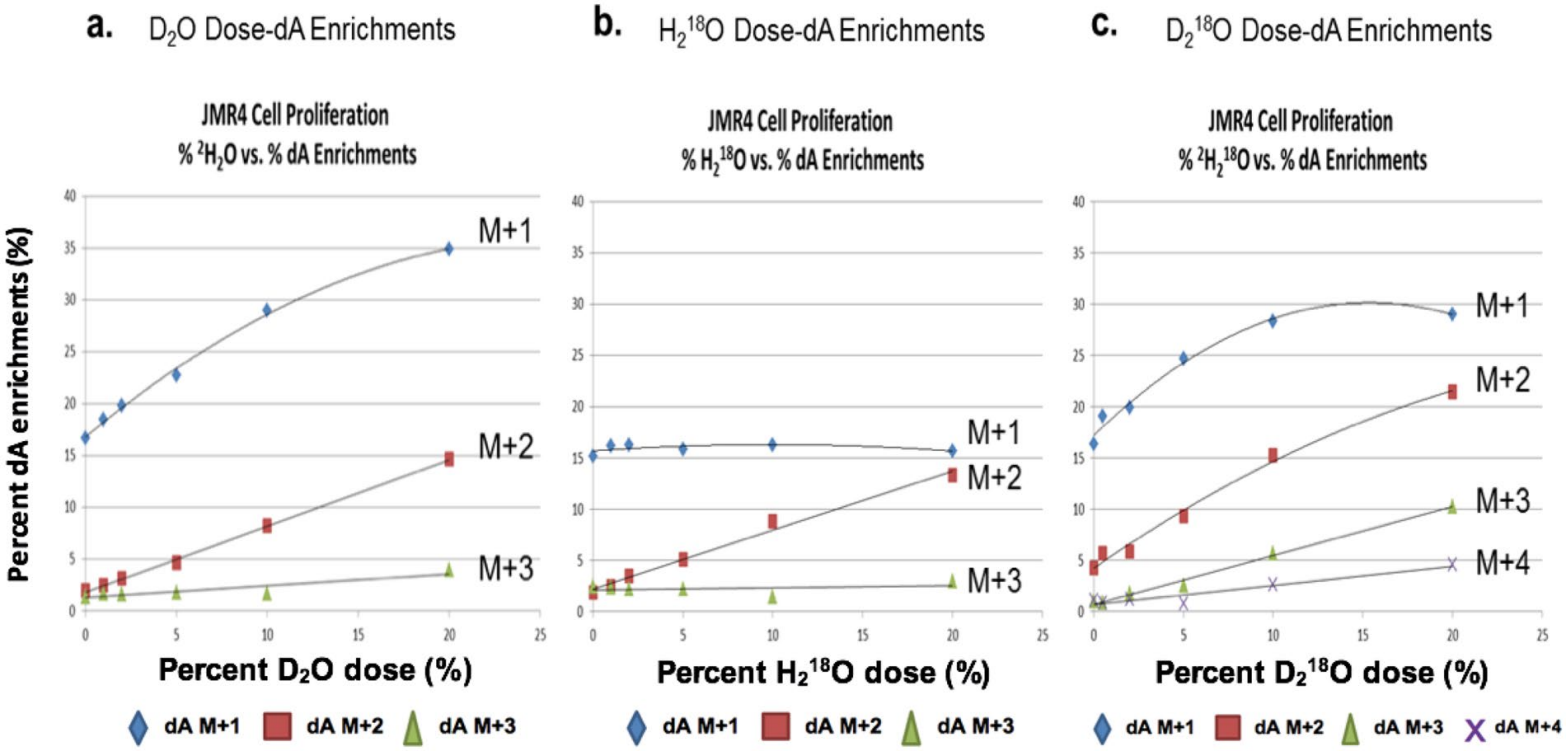
Heavy water dose-dA isotopic enrichments for mouse thymus tumor cell DNA dA M + 1, M + 2, M + 3 and M + 4 isotopologues after 4-day incubation. (**a**) Isotopic enrichments into the DNA dA isotopologues (M + 1, M + 2, M + 3) from mouse thymus tumor cells incubated in cell media fortified using 1 to 20% D_2_O, (v/v). (**b**) Isotopic enrichment into the DNA dA isotopologue (M + 2) from tumor cells incubated in cell media fortified using 1 to 20% H_2_^18^O, (v/v). (**c**) Isotopic enrichments into the DNA dA isotopologues (M + 1, M + 2, M + 3, M + 4) from tumor cells incubated in cell media fortified using 1 to 20% D_2_^18^O, (v/v).

A dose-dA isotopic enrichment study of the dA isotopologues was performed using mouse thymus tumor cells. Using dosing levels of 1, 2, 5, 10 and 20% (v/v) of D_2_O, H_2_^18^O, and D_2_^18^O, a dose-dA isotopic enrichment curve was constructed for each dA isotopologue (dA M + 1 to dA M + 4). As seen in Fig. 5a–c, different forms of stable heavy water have different profiles of isotopic enrichments into the dA isotopologues, with highest isotopic enrichments occurring in the dA M + 1 isotopologue, followed by enrichments into the dA M + 2 isotopologue. For dosing levels of 1–20% (v/v), as the dose of the stable heavy water increased, the isotopic enrichments into the individual dA isotopologues increased proportionally with nearly linear relationships.

### Heavy water dose-response effect on mouse thymus tumor cell proliferation (*in vitro*)

**Figure 6:**
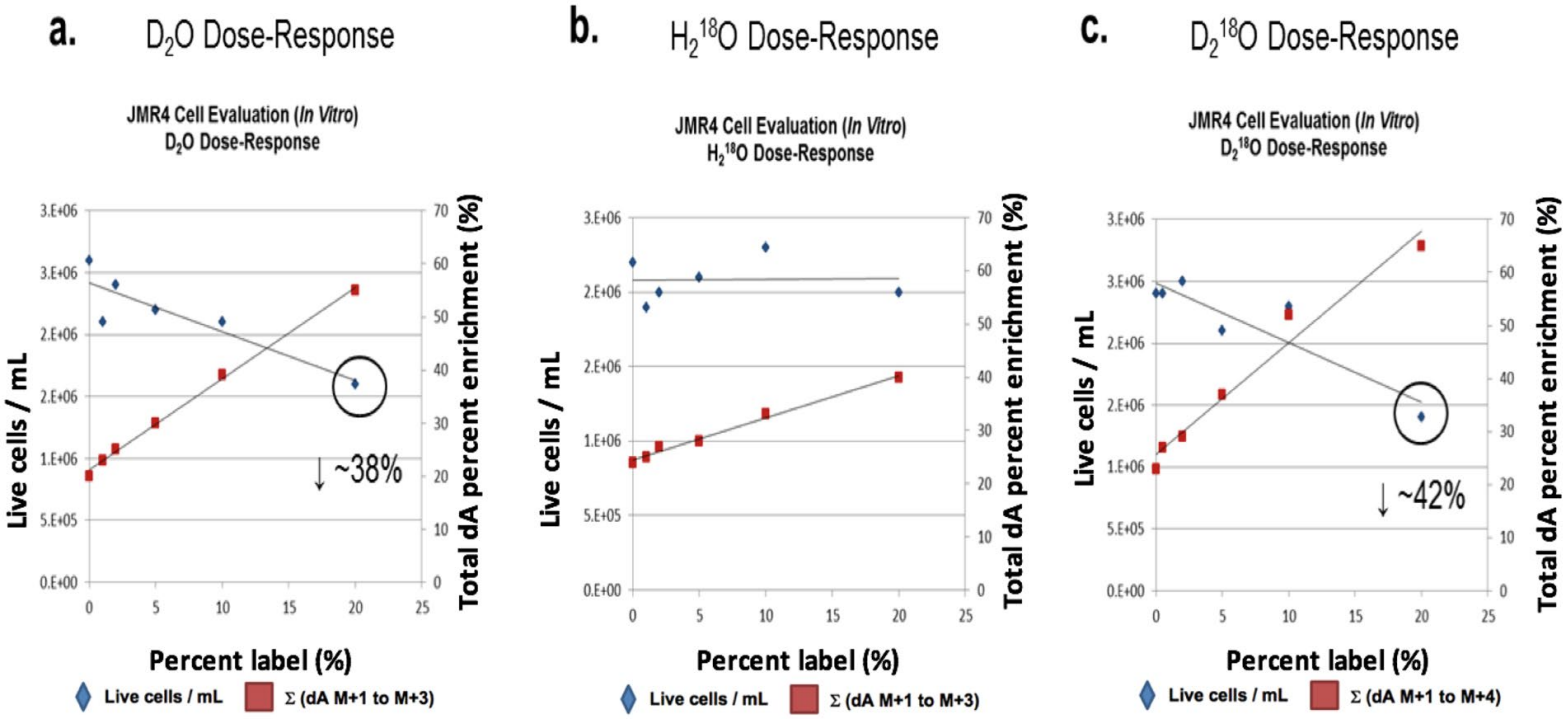
Heavy water dose-response effect on mouse thymus tumor cell viability (%) after 4-day incubation. (**a**) Total dA enrichments of mouse thymus tumor cells incubated in cell media fortified using 1 to 20% D_2_O (v/v) caused a reduction in proliferation and % cell viability at higher dosing levels (e.g. 20%, v/v). (**b)** Total dA enrichments of tumor cells incubated in cell media fortified using 1 to 20% H_2_^18^O (v/v) had no apparent effect on proliferation or % cell viability. (**c**) Total dA enrichments of tumor cells incubated in cell media fortified using 1 to 20% D_2_^18^O (v/v) caused a reduction in proliferation and % cell viability at higher dosing levels (e.g. 20%, v/v).

To investigate the potential effect of stable heavy water on mouse thymus tumor cell proliferation, we performed a dose-response study using dosing levels of 1, 2, 5, 10 and 20% (v/v) with D_2_O, H_2_^18^O, and D_2_^18^O. A dose-response curve was constructed comparing total isotopic enrichments into the dA isotopologues (summation of dA M + 1 to dA M + 4) versus cell count and viability (live cells/mL). As demonstrated in Fig. 6a–c, the total isotopic enrichments of the dA isotopologues (summation of dA M + 1 to dA M + 4) increase linearly in a dose-response manner, with D_2_O and D_2_^18^O water having the highest total enrichment in dA isotopologues. These two forms of stable heavy water had an inverse relationship between total isotopic enrichments into the dA isotopologues (summation of dA M + 1 to dA M + 4) versus cell count and viability (live cells/mL) with a decrease in cell proliferation of ~40% (Fig. 6a and c). The dose-response results for H_2_^18^O water revealed it had less total isotopic enrichments into the dA isotopologues (summation of dA M + 1 to dA M + 4) with no significant effect on cell count and viability (live cells/mL) (Fig. 6b).

### Method development of headspace-GC-NCI-MS for measurement of stable heavy water levels in total body water (TBW)

Knowing that higher levels of D_2_O or D_2_^18^O water may have potential cell modulation effects, we developed a simple and quantitative headspace-GC-NCI-MS method to accurately measure the stable heavy water levels in various biological matrices (e.g. cell media, urine, plasma, and saliva). Briefly, the method employs mass spectrometry using negative chemical ionization, as it is known to have very high sensitivity from the low background noise Supplementary Information, Fig. 5. The headspace method utilizes isothermal temperature (80 °C) and optimized basic conditions (e.g. 2 N NaOH) (Supplementary Information, Fig. 6) to facilitate the exchange of deuterium and ^18^O moieties from the stable heavy water to the acetone solvent (Supplementary Information, Figs 7 and 8).

The headspace method requires only 25 µL of the biological sample, 5 µL of 10 N NaOH, and 20 µL of acetone for analysis. After deuterium and ^18^O exchange, the isotopically labeled acetone (MW 58 – 1 [H+] = 57 m/z for negative chemical ionization (NCI) mode) was measured using headspace-GC-NCI-MS in full scan mode. Supplementary Information, Fig. 7 represents D_2_O standards in cell media (CM) and normalized MS profiles of hydrogen:deuterium (H:D) exchange to the acetone solvent. As the D_2_O standards increase in level, the acetone isotopic enrichment increases as demonstrated by NCI detection of the 58 (M + 1), 59 and 60 m/z ions. Quantitation of D_2_O in total body water was determined using the M + 1/M0 ratio (i.e. 58/57 m/z) with demonstrated linearity from 2.5–40% (v/v). Supplementary Information, Fig. 8 represents H_2_^18^O standards in CM and normalized MS profiles of ^16^O:^18^O exchange to the acetone solvent. Increasing levels of H_2_^18^O standards increased the isotopic enrichments into acetone, as demonstrated by NCI detection of the 59 ion (i.e. M + 2). Quantitation of H_2_^18^O in TBW was determined using the M + 2/M0 ratio (i.e. 59/57 m/z) with demonstrated linearity from 1–20% (v/v).

**Figure 7:**
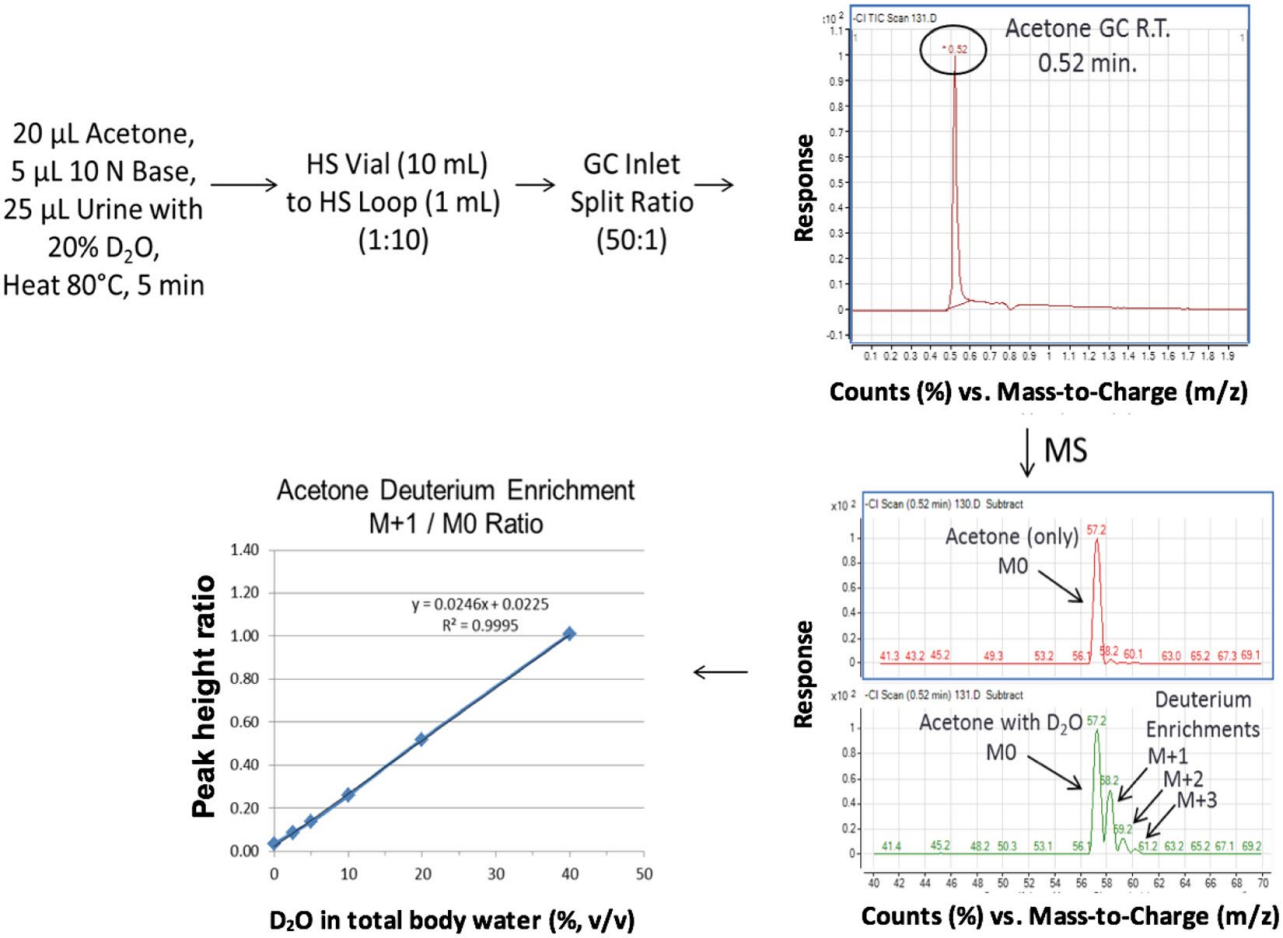
Test method diagram of rapid headspace-GC-NCI-MS full scan analysis with calibration curve of D_2_O in total body water (urine). Levels of D_2_O water (2–40%, v/v) in blank human urine were made basic (pH 13–14) using NaOH. Acetone was added, headspace vial capped and incubated using headspace method conditions (80 °C, 5 min) with analysis using headspace-GC-NCI-MS in full scan mode. Calibration curves were constructed using the acetone deuterium enrichment ratio (M + 1/M0) demonstrated good linearity (R^2^ = 0.9995) from 2–40%, v/v.

**Figure 8:**
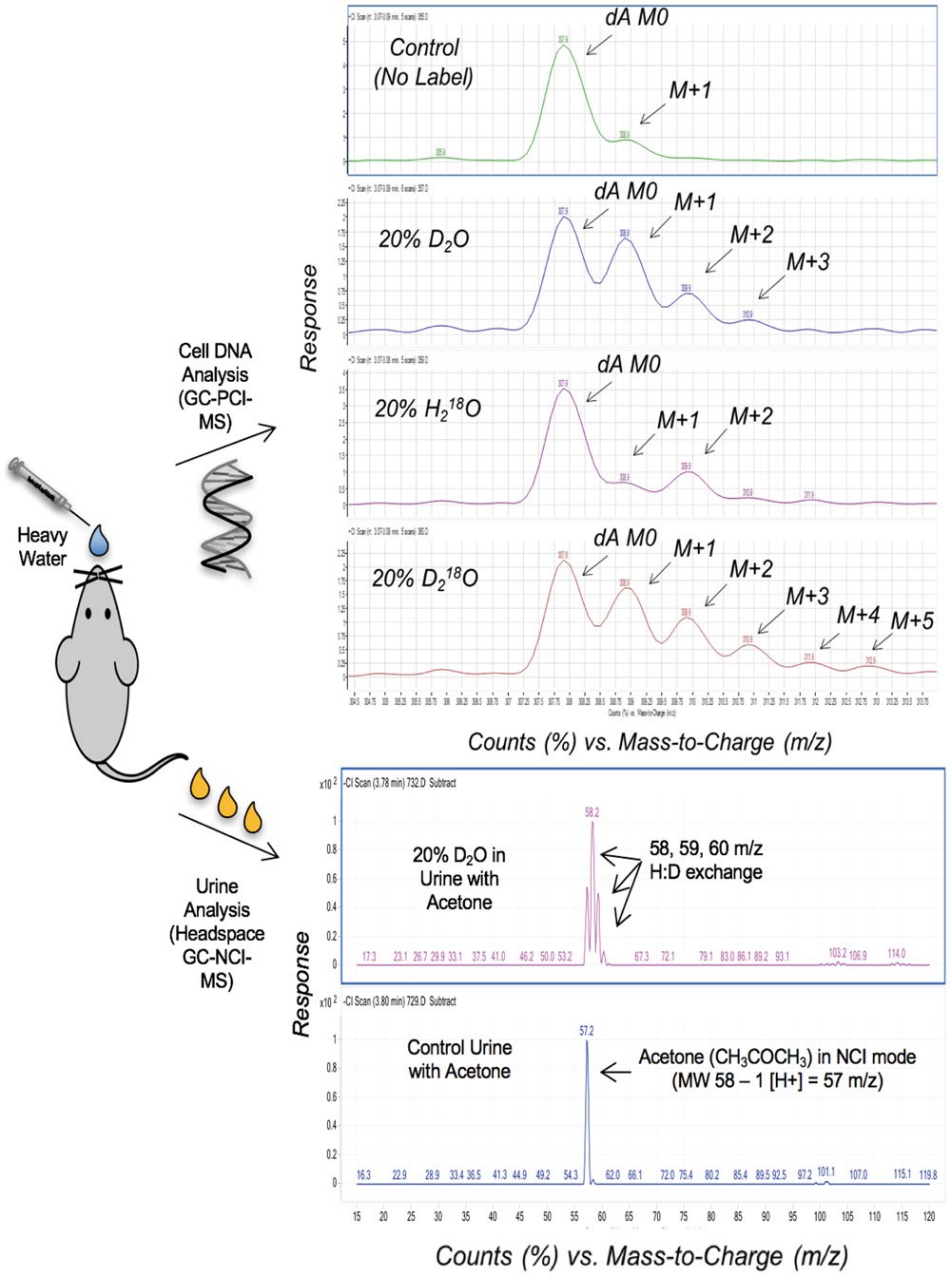
Mass spectrometry isotopic signatures (normalized) for stable heavy water in mouse cellular DNA and total body water (urine). After dosing with stable heavy water (e.g. D_2_O), DNA deoxyadenosine (dA) enrichments can be measured using GC-PCI-MS, with total body water measurements using headspace-GC-NCI-MS.

Since translational studies using stable heavy water labeling may include long-term maintenance dosing, a non-invasive TBW sample for monitoring and compliance testing would be beneficial to the clinical investigator. Urine collection is not invasive, thus urine collected was evaluated after fortifying samples with various amounts of the different forms of stable heavy water. As seen in Supplementary Information, Figure 9, D_2_O, H_2_^18^O and D_2_^18^O in mouse urine had unique MS profiles (i.e. isotopic fingerprints), based on the isotopic exchanges of hydrogen: deuterium (H: D) and ^16^O:^18^O from stable heavy water to the acetone solvent. Figure 7 depicts the new rapid test method and its utility for quantitating D_2_O in TBW (urine) with demonstrated MS calibration linearity from 2–40% (v/v). Figure 8 illustrates how the MS isotopic signatures (i.e. fingerprints) would be observed and measured in the DNA and TBW (e.g. 20% D_2_O in urine) following *in vivo* labeling with stable heavy water to evaluate mouse cell kinetics.

## Discussion

The evaluation of different forms of stable heavy water (e.g. D_2_O, H_2_^18^O, D_2_^18^O) for both basic and clinical research is important as they have been successfully used to study proteins, peptides, lipids, nucleic acids, metabolites, carbohydrates, and individual energy expenditures (i.e. D_2_^18^O). In addition, stable heavy water at lower doses (e.g. 5% TBW) is generally recognized as safe and can be used for translational studies involving human subjects^2^. Although D_2_O is the current gold standard and has been cited most in cell proliferation studies, our research suggests that H_2_^18^O may be a better isotopic label for such studies. By quantifying stable water isotopic enrichments into DNA deoxyadenosine (dA) using *in vitro* (mouse thymus tumor cell line) and *in vivo* (normal mouse bone marrow) experiments, we found that H_2_^18^O enrichment into the dA M + 2 isotopologue was better for labeling DNA in proliferating cells than either D_2_O or D_2_^18^O water. The use of H_2_^18^O offers better mass spectrometry (MS) quantitation of the DNA dA enrichments, and to our surprise, when dosing at higher levels (e.g. ≥20%, v/v), the mouse tumor cells with H_2_^18^O enrichments had a higher cell proliferation and better viability (live cells/mL) than those isotopically labeled using D_2_O or D_2_^18^O water.

An unexpected, yet important research observation, was that the higher levels of D_2_O and D_2_^18^O water (e.g. ≥20%, v/v) had significant negative modulatory effects on mouse tumor cell proliferation and viability. Other research groups have also reported negative cellular effects when using higher levels of D_2_O (e.g. 20%, v/v). One research group studied kangaroo kidney cells (*in vitro)* and high levels of D_2_O (75%, v/v), which caused negative effects on the formation of the mitotic spindles during cell replication^40^. Another research group studied the effects of D_2_O on a mouse solid tumor and reported that ~23–24 atom percent deuteration inhibited tumor growth; however, they were not able to state the mechanism of the deuterium affect presumably due to the resources available to scientist during this time (1950s)^41^.

More broadly taken, the negative modulatory effect on tumor cell proliferation may have important therapeutic implications. Anti-proliferative properties of these forms of stable heavy water, especially D_2_^18^O, may guide new strategies of treatment for malignant and/or infectious diseases, by slowing tumor or microbial growth, respectively. Currently, many treatments for diseases that are mediated by rapidly dividing cells aim to reduce cell proliferation; presumably, such conditions could benefit from the anti-proliferative properties of stable water isotopes, which are non-radioactive and tasteless. Our research also reports a linear relation between stable heavy water dosing and total isotopic enrichments into DNA, which may cause instability in the DNA moiety at higher levels of D_2_O and D_2_^18^O, thus affecting cell proliferation. When comparing D_2_O and D_2_^18^O water results, total isotopic enrichments were higher with D_2_^18^O water, presumably from isotopic enrichments of both the deuterium and ^18^O moieties into the dA isotopologues.

Our research also indicates that the deuterium isotopic enrichments into the dA isotopologues may be reversible, unlike current cancer treatments using alkylating agents (e.g. cyclophosphamide), which are known to have adverse side effects from non-specific and irreversible binding to the DNA moiety. Our research offers new knowledge that each DNA nucleoside is isotopically enriched by stable heavy water and the isotopic enrichments are dose-related and reversible. Combining these findings with the knowledge that there are more than 5 billion nucleotides in the diploid human genome^42^, it is reasonable to hypothesize that higher dosing levels (e.g. ≥20%, v/v) of D_2_O and D_2_^18^O water could alter DNA in such a way that a proliferating cell (e.g. tumor) cannot replicate itself. Water has a very long half-life in the human body (>7 days)^43^. It rapidly diffuses throughout body water and tissues (high V_D_). Water is not metabolized (phase I/phase II) and is primarily cleared via kidneys. These factors support the need for further investigations of stable water isotopes and their therapeutic potential for modulating cell proliferation, which may include its use in conjunction with currently available cancer treatments. Our future research will focus on elucidating the mechanism-of-action for the negative impact of higher levels of D_2_O and D_2_^18^O water on tumor cell proliferation/viability and will include evaluation using human cancer cell lines.

We also report the development of a quantitative method to measure stable heavy water levels in total body water (TBW). For translational research involving human subjects, knowing the TBW levels of stable heavy water is important, especially given the relationship between stable heavy water dose and DNA isotopic enrichments described here, and given our findings that higher levels of D_2_O or D_2_^18^O may have negative effects on cell proliferation and cell viability. The basis of our new test method is a rapid gas phase isotopic exchange of the H:D and ^16^O:^18^O moieties between stable heavy water and the acetone solvent, which occurs during the headspace incubation (i.e. 80 °C for ~5 min). The simple and rapid headspace-GC-NCI-MS method has recently been utilized for our translational research, with an analytical run time of 1 min and requiring only 25 µL of urine, saliva, blood, or cell media for analysis.

## Methods

### Chemicals, Reagents, Gases, and Supplies

Deuterium oxide (D2, 99.8%), H_2_^18^O (18 O, 97%), and ^2^H_2_^18^O (D2, 98%; 18 O, 97%) were purchased from Cambridge Isotope Laboratories (Andover, MA). Methanol (high purity) was purchased from Honeywell Burdick & Jackson^®^ (Muskegon, MI). Acetone (HPLC grade) and sodium hydroxide (10 N, 30% w/w, Certified) were purchased from Fisher Scientific (Fairlawn, NJ). MethElute™ derivatization reagent (0.2 M Trimethylanilinium hydroxide in methanol, (pH ≥ 10) was purchased from Thermo Scientific (Waltham, MA) and used as received. Oasis^®^ HLB µElution plates (30 µM) were purchased from Waters Corporation (Milford, MA). GC headspace vials (10 mL) and high temperature screw top caps were purchased from Agilent Technologies (Santa Clara, CA). Standard material of 2′-deoxyadenosine monohydrate (purity > 99%) was purchased from Sigma-Aldrich (St Louis, MO). Gases used for GC-MS/MS analysis were helium (Grade 5.5 purity), nitrogen (Ultra high purity), isobutane (Matheson 99.99% purity) and methane (Research Grade purity) and were purchased from Roberts Oxygen Company (Rockville, MD). Gas purifiers purchased from Agilent Technologies were used to remove hydrocarbons and moisture from the gases.

### Equipment and PC Workstation Software

The extraction plate manifold used for Oasis 96-well plate HLB extractions was purchased from Waters Corporation (Milford, MA). The Savant DNA110 SpeedVac^®^ used for concentrating samples was purchased from Thermo Fisher Scientific (Waltham, MA). The Agilent 7890 A GC, LTM Series II Fast GC Module, 7000 A GC-MS/MS Triple Quadrupole, 7693 Autosampler and 7697 A Headspace Sampler were purchased from Agilent Technologies. For GC-MS/MS data acquisition and processing, Agilent MassHunter workstation software included Acquisition, Qualitative and Quantitative Analysis.

### Preparation of Calibration and Reference Solutions

A stock solution was prepared containing deoxyadenosine (dA). The stock solution [~1 mg/mL] was prepared in methanol and stored in polypropylene bottles at 4 °C. A working reference solution was prepared daily by appropriate dilution of the stock solution with MethElute™ (1:1, v/v), and stored in a deactivated glass autosampler vial at room temperature. The reference solution was used to monitor GC component peak shape and MS response factor.

A solid phase extraction (SPE) QC stock solution, used to monitor the SPE efficiency, was prepared containing ~1 mg/mL level of deoxyadenosine (dA). The QC stock solution was prepared in methanol and stored in a polypropylene bottle at 4 °C. A QC working reference solution was prepared by appropriate dilution of the stock solution with phosphate buffered saline, and stored in a polypropylene bottle at 4 °C.

For headspace analysis, the calibration standards and biological samples (e.g. urine) were prepared directly into the GC headspace vials (Supplementary Information, Table 1).

### *In Vitro* Mouse Thymus Lymphoma Cell Cultures

The cell media consisted of 1000 mL of 1x Dulbecco’s Modified Eagle’s Medium (DMEM), which contained 4500 mg/L D-glucose, 584 mg/L L-glutamine, and 110 mg/L sodium pyruvate (Invitrogen, Thermo Fisher). The DMEM was supplemented with 10 mL of 100x Pen-Strep Glutamine containing penicillin, streptomycin, and L-glutamine (Invitrogen). Additionally, 10 mL of non-essential amino acids (NEAA, 10 mM, Invitrogen), 100 mL of heat inactivated Fetal Calf Serum (Hyclone), 10 mL of sodium pyruvate (100 mM, Invitrogen), and 1 mL of 2-mercaptoethanol (55 mM, Invitrogen) were mixed into the cell media. The modified cell media was filtered using a Rapid-Flow Nalgene filter (Thermo Scientific), and was stable for up to 6 months when stored at 4 °C.

The JMR4 tumor cells were cloned from the JMR cell line, which was derived from a thymic lymphoma originating in a 2 C TCR transgenic mouse. The JMR4 cells were characterized as a CD4 + thymocyte-like cell line expressing the 2 C TCR. The tumor cells (suspension) were cultured in Corning^®^ 25 cm^2^ sterile cell culture flasks, split every 4–5 days using a 1:10 split ratio with cell seeding density of ~300,000 cells/mL, and maintained in a Heracell^®^ 150 incubator set at 10% CO_2_, ≥95% relative humidity, and 37 °C.

### *In Vivo* Mouse Bone Marrow Collection

Female BALB/cAnNCr (H-2^d^) mice were purchased from Charles River, Wilmington, MA. Mice received regular drinking water and food until stable water isotope labeling commenced. The NIH NCI Animal Care and Use Committee approved all animal protocols. Stable water isotope labeling was performed according to our previously published protocol (D. Farthing *et al*., 2013). Briefly, 8% (v/v) of the stable heavy water isotope (D_2_O or H_2_^18^O) was provided in drinking water for 7 days, following an initial intraperitoneal bolus (~700 µL) of saline (0.9%, wt/v) made using 100% of the stable water isotope. Urine was collected from each mouse on day + 7 and stored at −20 °C until MS analysis of the stable heavy water level. Bilateral femur and tibia from each mouse were collected for bone marrow extraction. Each bone fragment was flushed with mouse media (RPMI Media (Gibco) supplemented with 10% v/v fetal bovine serum (HyClone), 1X penicillin/streptomycin/glutamine (Gibco), 1 mM sodium pyruvate (Gibco), 1X MEM Non-Essential Amino Acids (Gibco), and 1X β-mercaptoethanol (Gibco)).

Single cell suspensions were obtained for each bone marrow sample, followed by red blood cell lysis using ACK lysis buffer (Lonza). Cells were counted using Nexcelom Cellometer Auto T4 (Life Technologies, Grand Island, NY) and Trypan Blue 0.4% v/v in PBS (Lonza). Following cell counting, each bone marrow sample was centrifuged for 5 minutes at 480 × g (1,500 RPM) at 4 °C; resulting cell pellets were re-suspended in phosphate buffered saline (Gibco). Multiple aliquots of 100,000 cells each were made for each bone marrow sample. Samples were stored at −80 °C until DNA extraction and MS analysis.

### Cell Lysis, DNA Extraction, and Hydrolysis

Collected cells were lysed using the EpiSonic™ Multi-Functional Bioprocessor Model 1100 (Epigentek Group, Farmingdale, NY) (Supplementary Information, Procedure for Episonic™ Sonoporation of Cells), and the DNA was hydrolyzed into its nucleoside bases using a commercially available kit (Epigentek EpiQuik^™^). The kit contains DNA hydrolysis enzymes and digestion buffer, and was used as received. Following 2-hour incubation at 37 °C and gentle mixing using an Eppendorf Thermomixer R, the enzymes provide DNA hydrolysis efficiencies of 60–90%.

### Deoxynucleoside Purification and Derivatization

Solid phase extraction (SPE) was utilized to remove sample buffer salts, water, and to concentrate the sample prior to GC-MS/MS analysis. The DNA hydrolysis and SPE procedure is listed in Supplementary Information, Procedure of DNA Hydrolysis, and Oasis HLB Solid Phase Extraction (SPE) of Deoxyadenosine (dA).

### Headspace, GC, and MS Instrument Conditions

Supplementary Information, Set-points for Agilent Equipment lists the headspace sampler, GC and MS instrument conditions used for deoxyadenosine and total body water analysis.

### Computations

For all computations, MS data collected using PCI or NCI modes of MS were processed using Agilent MassHunter Qualitative and Quantitative software. For dA and its isotopologues (e.g. dA M + 1, dA M + 2), relative peak area computations, regression analysis, x-y graphs and bar charts were performed using Agilent MassHunter and MS Excel software.

## Electronic supplementary material

Uncharted Waters – Comparing stable isotopic forms of heavy water incorporation into the DNA of proliferating cells
