# Supplementary material for: Comparing DNA enrichment of proliferating cells following administration of different stable isotopes of heavy water
Source: Sci Rep. 2017 Jun 22;7:4043. doi: 10.1038/s41598-017-04404-2 (PMC5481421; doi:10.1038/s41598-017-04404-2)
Supplement: Supplementary file 1 — Uncharted Waters – Comparing stable isotopic forms of heavy water incorporation into the DNA of proliferating cells [file 41598_2017_4404_MOESM1_ESM.docx]

**Supplementary Information**

**Comparing DNA enrichment of proliferating cells following administration of different stable isotopes of heavy water**

**Authors:**

Don E. Farthing NIH, NCI, ETIB, Bethesda, MD

Nataliya P. Buxbaum, NIH, NCI, ETIB, Bethesda, MD

Philip J. Lucas, NIH, NCI, ETIB, Bethesda, MD

Natella Maglakelidze, NIH, NCI, ETIB, Bethesda, MD

Brittany Oliver, NIH, OCRT&ME, Bethesda, MD

Jiun Wang, NIH, NCI, ETIB, Bethesda, MD

Kevin Hu, NIH, NCI, ETIB, Bethesda, MD

Ehydel Castro, NIH, NCI, ETIB, Bethesda, MD

Catherine V. Bare, NIH, NCI, ETIB, Bethesda, MD

Ronald E. Gress, NIH, NCI, ETIB, Bethesda, MD

**Table of Contents:**

1. Supplementary Information, Figure 1: Histogram of Episonic™ pulsed sonoporation on JMR4 mouse thymus tumor cells – Page 3
2. Supplementary Information, Figure 2: Cell image of Episonic™ pulsed sonoporation of JMR4 mouse thymus tumor cells – Page 4
3. Supplementary Information, Figure 3: Reproducibility of cell sample preparation for analysis of stable heavy water enrichment into DNA deoxyadenosine (dA). *In vitro and in vivo* results of stable heavy water enrichments into the dA isotopologues – Page 5
4. Supplementary Information, Figure 4. JMR4 mouse thymus tumor cells incubated using different forms of stable heavy water – Page 6
5. Supplementary Information, Figure 5. Negative chemical ionization background counts from isobutane reagent gas – Page 7
6. Supplementary Information, Figure 6: Effect of sodium hydroxide level on hydrogen: deuterium exchange to acetone – Page 8
7. Supplementary Information, Figure 7: Mass spectrometry profiles of hydrogen:deuterium isotopic exchange for increasing levels of D_2_O (v/v) in cell media to the acetone solvent – Page 9
8. Supplementary Information, Figure 8. Mass spectrometry profiles of ^16^O:^18^O isotopic exchange for increasing levels of H_2_^18^O (v/v) in cell media to the acetone solvent – Page 10
9. Supplementary Information, Figure 9. Mass spectrometry isotopic signatures (normalized) for different forms of heavy water in TBW (mouse urine) – Page 11
10. Supplementary Information, Table 1: Preparation of D_2_O heavy water standards and urine samples for headspace analysis – Page 12
11. Procedure for Episonic™ Sonoporation of Cells – Page 13
12. Procedure for DNA Hydrolysis and Oasis HLB Solid Phase Extraction (SPE) of Deoxyadenosine (dA) – Page 14
13. Set-points for Agilent 7890A GC, 7693 Autosampler and 7000B MS Triple Quad for dA Analysis – Pages 15 and 16
14. Set-points for Agilent 7697A Headspace Sampler – Page 17
15. Set-points for Agilent 7890A GC and 7000B MS Triple Quad for Total Body Water (TBW) Analysis – Pages 18 and 19


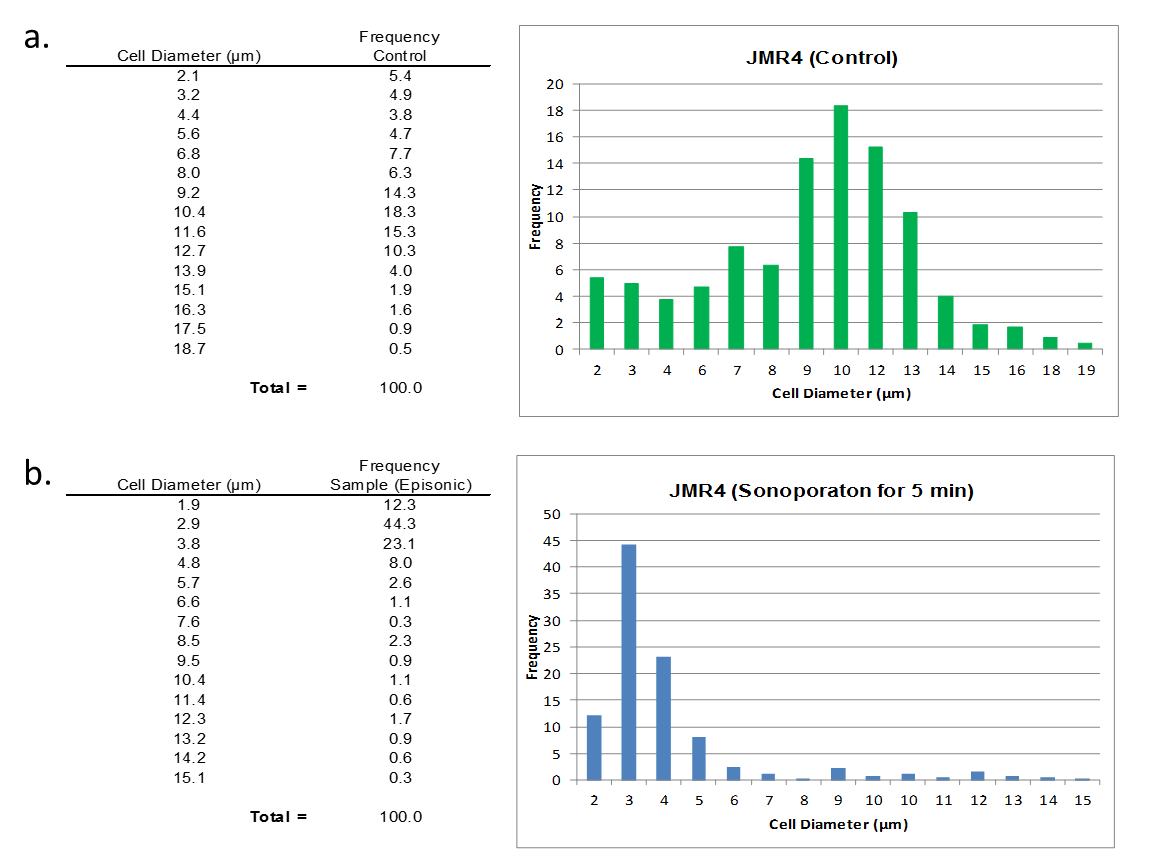


**Supplementary Information, Figure 1 | Histogram of Episonic™ pulsed sonoporation on JMR4 mouse thymus tumor cells.** JMR4 mouse thymus tumor cells **(a)** before (control) and **(b)** after pulsed sonoporation (i.e. pulse-on 20 sec., pulse-off 10 sec., 5 min sonoporation process time). Cell diameter (µm) and frequency were measured using the Cellometer^®^ Auto T4 Cell Viability Counter.


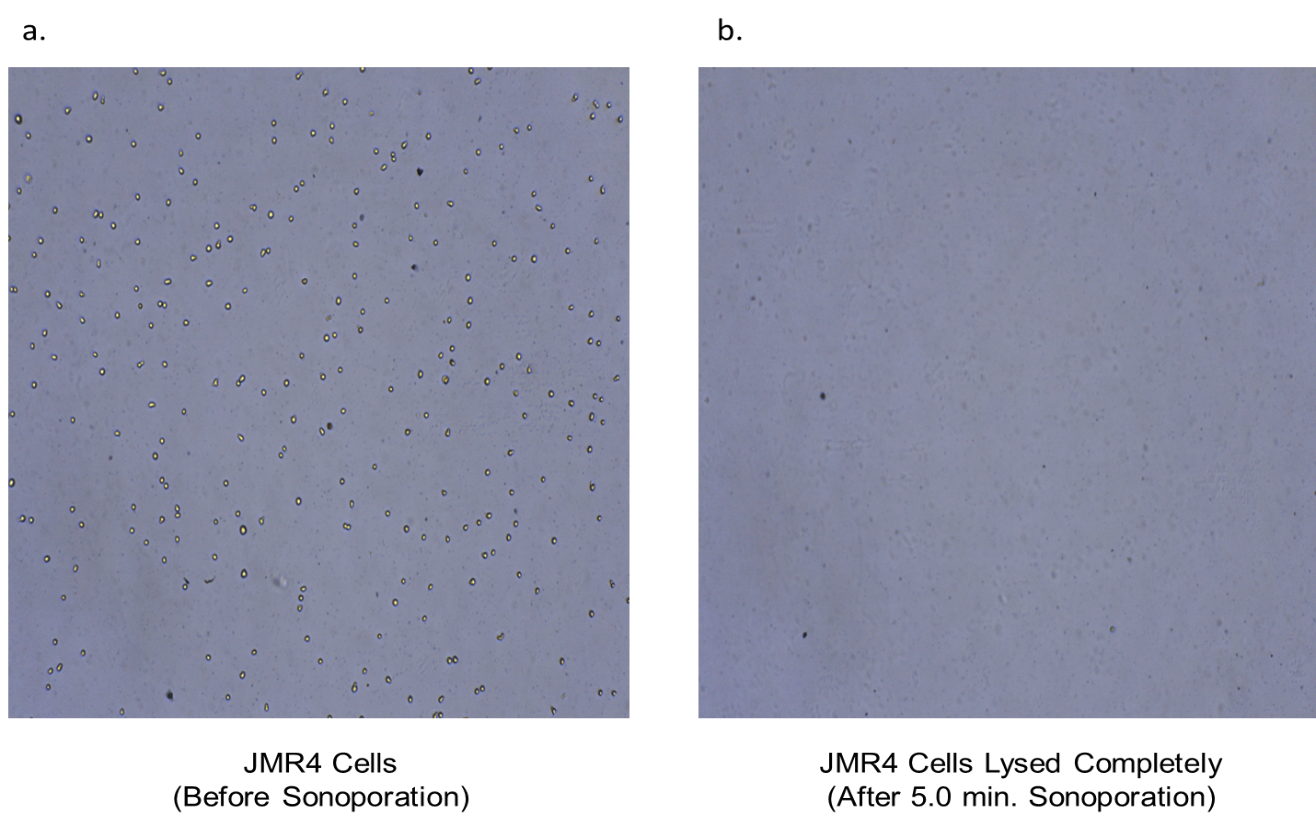


**Supplementary Information, Figure 2 | Cell image of Episonic™ pulsed sonoporation of JMR4 mouse thymus tumor cells.** JMR4 mouse thymus tumor cells **(a)** before (control) and **(b)** after pulsed sonoporation (i.e. pulse-on 20 sec., pulse-off 10 sec., 5 min sonoporation process time). Cell images were taken with the Cellometer^®^ Auto T4 Cell Viability Counter using the same image resolution.


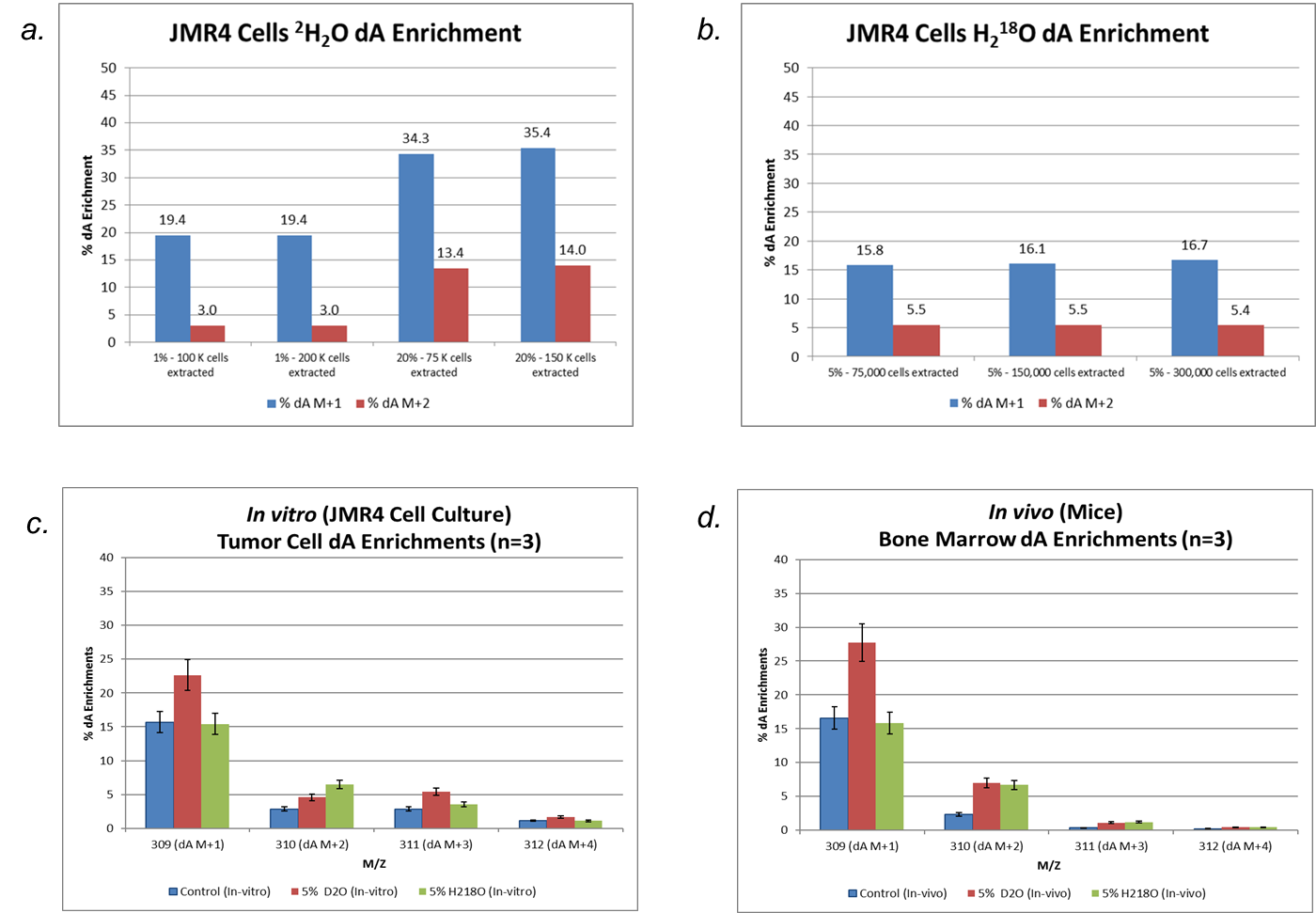


**Supplementary Information, Figure 3 (a, b) | Reproducibility of cell sample preparation for analysis of stable heavy water enrichment into DNA deoxyadenosine (dA). Figure 3 (c, d) | *In vitro and in vivo* results of stable heavy water enrichments into the dA isotopologues.** The isotopically labeled cells were lysed, DNA hydrolyzed to its base pairs, and the dA extracted/purified for evaluation by GC-PCI-MS/MS. To evaluate extraction reproducibility, i*n vitro* JMR4 mouse thymus tumor cells were dosed at various concentrations (e.g. 1, 5, and 20%, v/v) of either (**a**) D_2_O or (**b**) H_2_^18^O and various amounts (e.g. 75,000, 300,000) of cells were extracted for analysis. (**c**) *In vitro* experiments (n=3) for dA enrichments of the mouse thymus tumor cells incubated in cell media fortified using either 5% D_2_O or 5% H_2_^18^O (v/v). (**d**) *In vivo* experiments (n=3) for dA enrichments of mouse bone marrow cells after dosing healthy normal mice to ~5% D_2_O or ~5% H_2_^18^O (v/v) of their total body water (TBW).


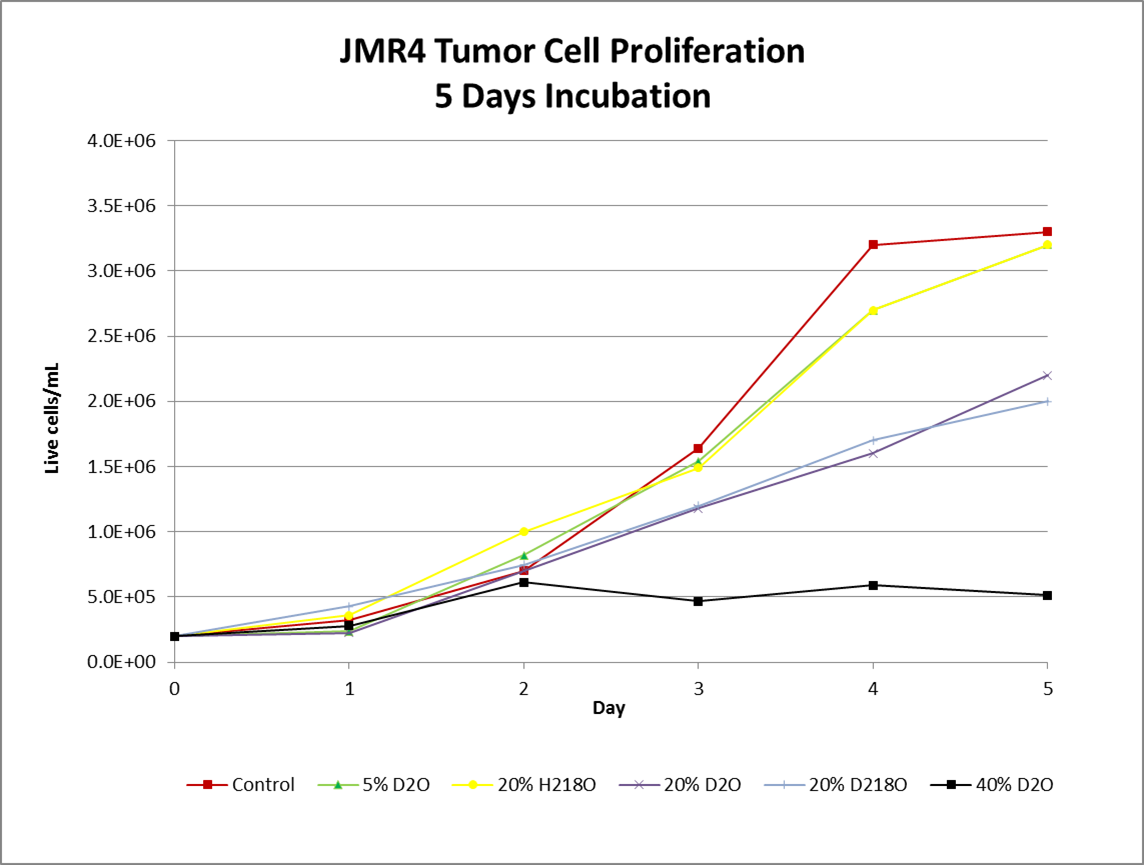


**Supplementary Information, Figure 4 | JMR4 mouse thymus tumor cells incubated using different forms of stable heavy water.** The mouse thymus tumor cells were dosed using various levels of D_2_O (0, 5, 20 and 40%, v/v), H_2_^18^O (20%, v/v), or D_2_^18^O (20%, v/v), and were incubated in cell media (without replacement) for 5 days at 10% CO_2_, >95% relative humidity and 37°C. The mouse thymus tumor cells dosed using higher levels of deuterium (e.g. ≥20%) had reduced rates of cell proliferation by the 3rd day of incubation. Small aliquots (50 µL) were taken for each time point measurement and cell counting was performed using the Cellometer™ Auto T4 Cell Counter.


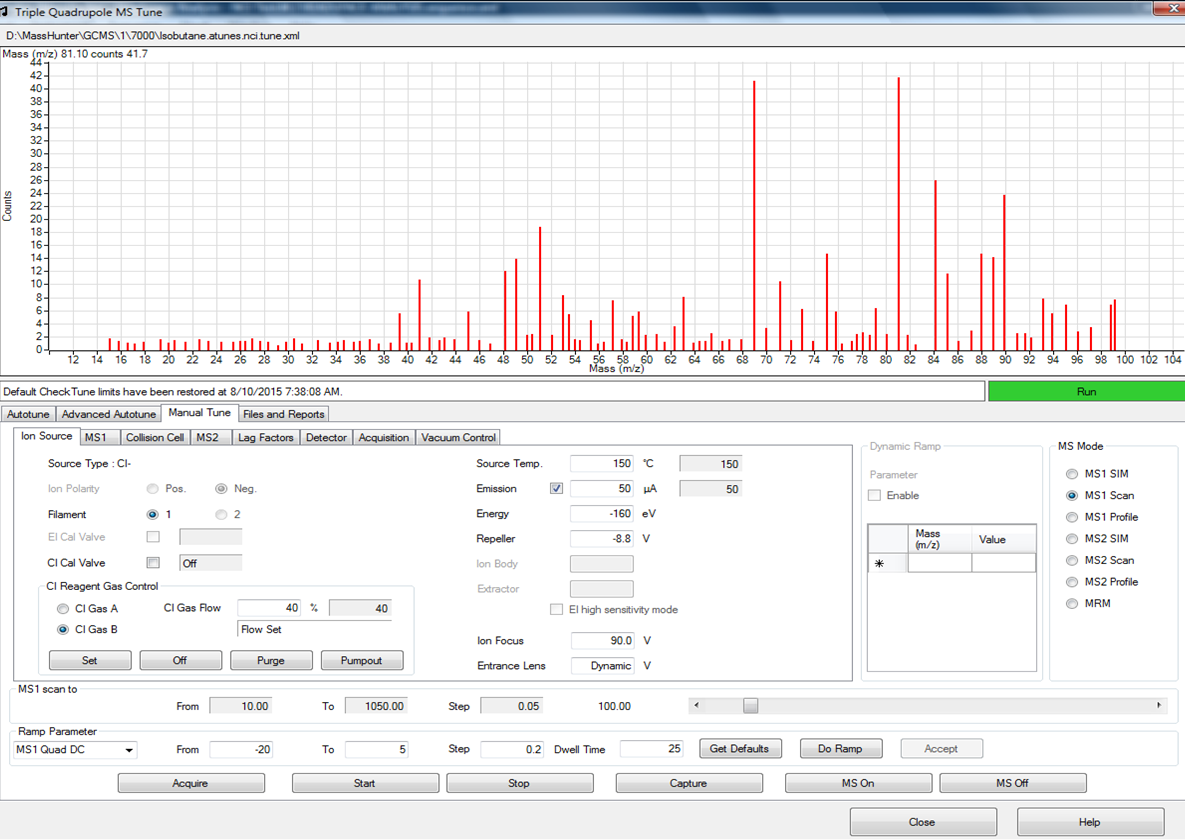


**Supplementary Information, Figure 5 | Negative chemical ionization background counts from isobutane reagent gas.** Full scan mass spectrum of isobutane reagent gas used in the negative chemical ionization (NCI) mode of MS. The extremely low background counts in the mass spectrum allows for high method sensitivity (i.e. signal/noise).


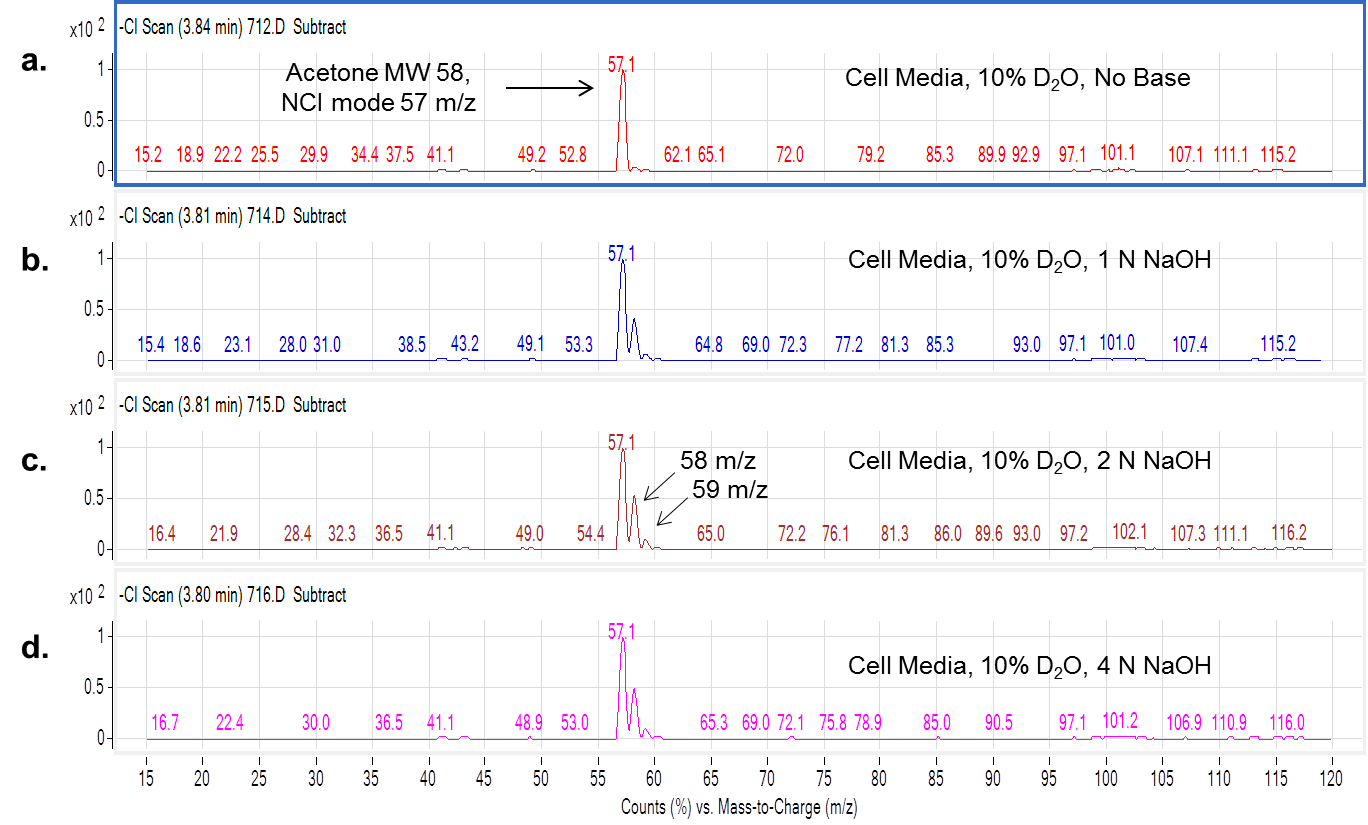


**Supplementary Information, Figure 6 | Effect of sodium hydroxide level on hydrogen: deuterium exchange to acetone.** Levels of sodium hydroxide (NaOH) were increased, which increased the hydrogen: deuterium (H:D) exchange to the acetone moiety. Acetone (MW 58) has a mass of 57 m/z after proton (H^+^] abstraction, when operating in the negative chemical ionization (NCI) mode of MS. Using cell media and 10% D_2_O, levels of NaOH base were evaluated: **(a)** No added NaOH, **(b)** 1 N NaOH, **(c)** 2 N NaOH and **(d)** 4 N NaOH. Increases in the 58 and 59 m/z ions indicate that strongly basic conditions (e.g. pH 13-14) favor H:D exchange under the GC-headspace method conditions (80ºC with 5 min incubation).


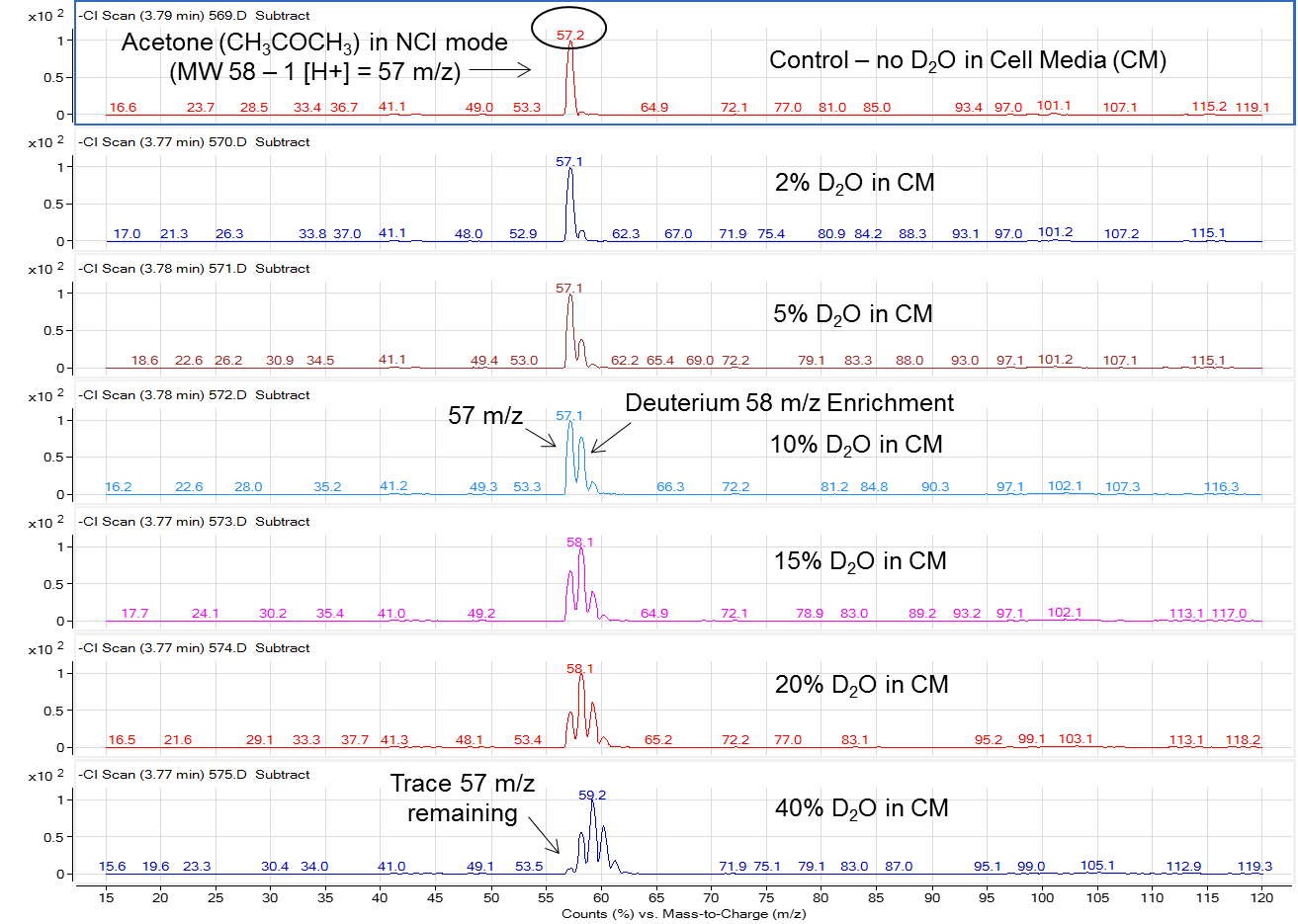


**Supplementary Information, Figure 7 | Mass spectrometry profiles of hydrogen: deuterium isotopic exchange for increasing levels of D_2_O (v/v) in cell media to the acetone solvent.** Levels of D_2_O (2-40%, v/v) in cell media (CM) were adjusted to basic pH (e.g. pH 13-14) using NaOH. Acetone was added with evaluation under GC-headspace method conditions (Isothermal at 80ºC, 5 min incubation). Mass increases in the 58, 59, 60 and 61 m/z ions demonstrate additional H:D exchanges at the higher levels of D_2_O in cell media.


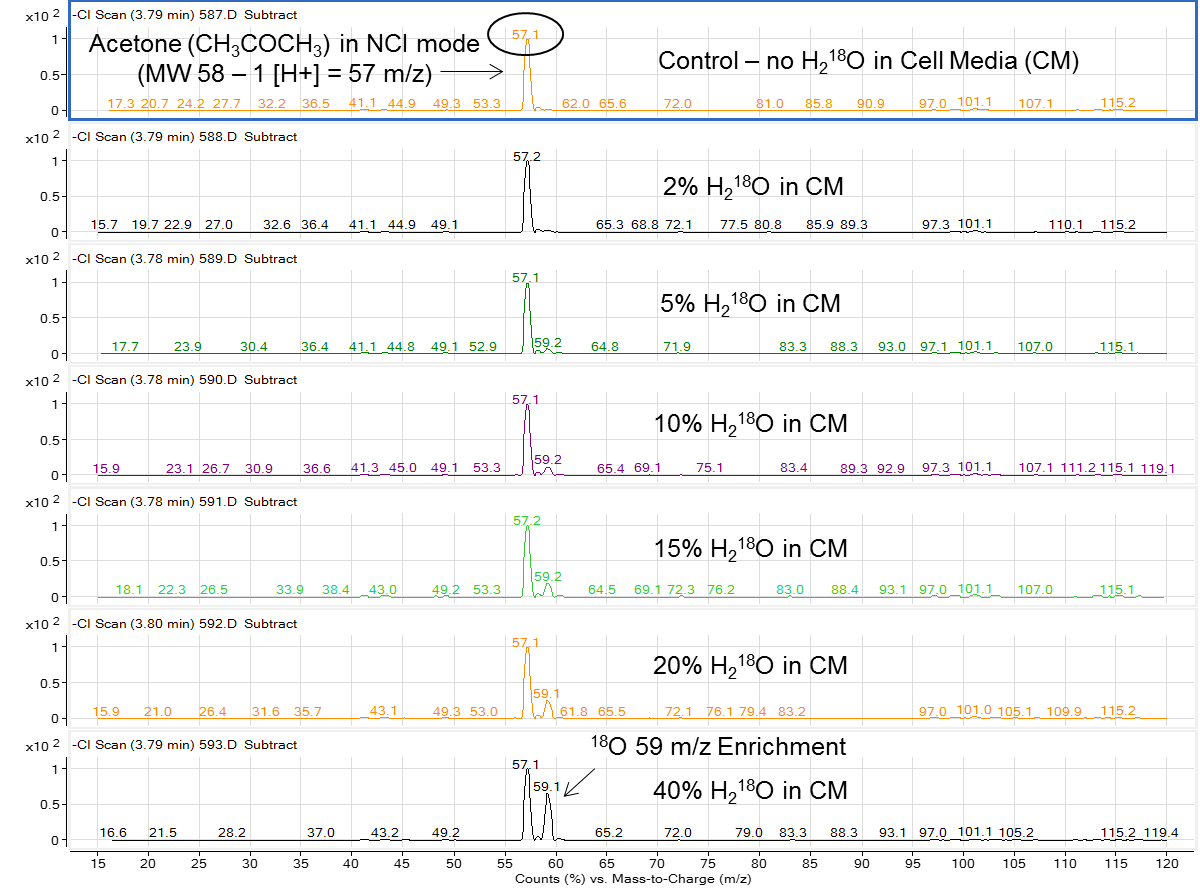


**Supplementary Information, Figure 8 | Mass spectrometry profiles of ^16^O:^18^O isotopic exchange for increasing levels of H_2_^18^O (v/v) in cell media to the acetone solvent.** Levels of H_2_^18^O (^18^O) water (2-40%, v/v) in cell media (CM) were made basic (e.g. pH 13-14) using NaOH. Acetone was added with evaluation under GC-headspace method conditions (80ºC with 5 min incubation). Mass increases in the 59 m/z ion demonstrate the ^16^O:^18^O exchange from increases in the levels of fortified H_2_^18^O water.


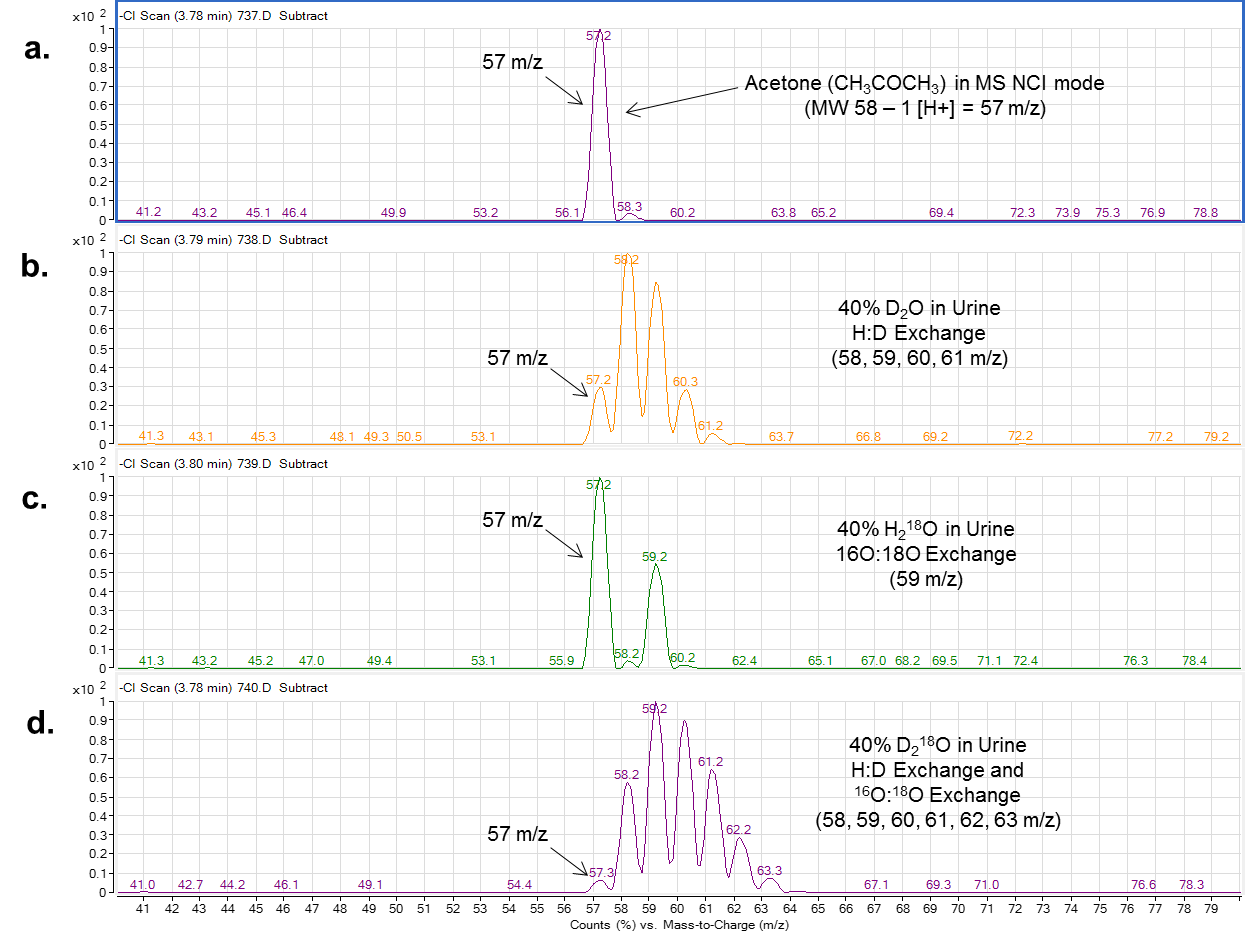


**Supplementary Information, Figure 9 | Mass spectrometry isotopic signatures (normalized) for different forms of heavy water in TBW (mouse urine).** (**a**) Headspace-GC-NCI-MS mass spectrum of acetone solvent. Acetone (MW 58) loses a proton in the negative chemical ionization (NCI) mode of MS, resulting in a parent ion of 57 m/z. (**b**) H: D exchange between a mouse urine level of D_2_O (40%, v/v) and acetone. (**c**) ^16^O:^18^O exchange between a mouse urine level of H_2_^18^O (40%, v/v) and acetone. (**d**) H:D and ^16^O:^18^O exchanges between a mouse urine level of D_2_^18^O (40%, v/v) and acetone.

**Supplementary Information, Table 1**

**Preparation of D_2_O Heavy Water Standards and Urine Samples for Headspace Analysis**

**Notes:**

1. Use Agilent 10 mL screw top headspace vials and caps with high temperature septa (low bleed).
2. The final pH (basic) of the headspace sample mixture is 13-14.

**Procedure for Episonic™ Sonoporation of Cells**

EpiSonic™ Multi-Functional Bioprocessor Model 1100

1. Set chiller to 4ºC (bath reservoir ~6-8ºC).
2. Pipette ~50 µL of cell suspension into 1.5 mL polypropylene micro-centrifuge tube (thick wall). Caution, the sonicator can easily crack thin wall sample tubes.
3. Prior to sonoporation, allow cells in tubes to cool in the water bath reservoir ~10 min.
4. Set pulse amplitude (A) to 14 or higher (based on targeted watts); Target pulse amplitude to deliver ~140-160 watts during cell sonoporation.
5. Set pulse-on to 20 sec.
6. Set pulse-off to 10 sec.
7. Set process time to 5 min.
8. Episonic calculated total time of cell sonoporation is 7.5 min.
9. Episonic calculated duty cycles are 15.
10. Record total kilo-joules (kJ) of work performed.
11. After sonoporation, the cells are completely lysed with some DNA shearing.

Note: It is important to avoid the micro centrifuge tube bottom touching the Episonic sonication horn, as it can crack the tube during sonication. It is also important to note that as the Episonic sonication horn ages from use, it may be necessary to increase the amplitude (A) to deliver the targeted wattage to completely lyse the cells.

**Procedure for DNA Hydrolysis and Oasis HLB Solid Phase Extraction (SPE) of Deoxyadenosine (dA)**

DNA Hydrolysis (EpiQuik^™^ One-Step DNA Hydrolysis Kit containing DH1, DH2 and DH3)

1. Pipette 43 µL DH3 (buffer) into 1.5 mL plastic micro-centrifuge tube.
2. Pipette 50 µL cells or DNA standard material (~500 ng).
3. Pipette 1 µL DH1 (enzymes) and 1 µL DH2 (enhancer).
4. Cap and mix gently.
5. Gently mix micro-centrifuge tube (Eppendorf Thermomixer R, 300 rpm, 37°C, 1-2 hrs.). Do not incubate for more than 2 hours as reaction is reversible. Alternatively, the enzymatic reactions can be stopped by incubating at 95°C for 10 min.
6. Centrifuge 500 x g, 25°C for 1 min to bring down cap condensate (if present).

Note: Limit for EpiQuik DNA hydrolysis is ~1,000 ng DNA (i.e. ~125,000 mouse cells or ~170,000 human cells). EpiQuik^™^ enzymes should be stored frozen at -20°C. After thawing, enzymes can be stored at 4°C for 1 week, or refrozen at -20°C. The vendor states that the enzymes can be freeze-thawed twice without significant loss in activity. If storing refrigerated, enzymes will lose activity over a period of time (e.g. several weeks).

Solid Phase Extraction (Oasis^®^ HLB µElution 96 Well Plate, 30 µm particle, 2 mg HLB Sorbent)

**HLB Sorbent Conditioning**

1. Pipette ~150 µL MeOH; purge using ~4-6 inches Hg.

2. Pipette ~250 µL PBS buffer (1X, pH 7.4); purge using ~4-6 inches Hg.

**Sample Application**

1. For DNA hydrolysis sample, pipette 25 µL I.S. into sample tube, mix well.
2. Transfer and rinse tube using 2 x 110 µL PBS.
3. Add 300 µL PBS into HLB sample well plate (total well volume ~500 µL).
4. For QC sample, pipette 25 µL QC solution and add 500 µL PBS.
5. For best component recoveries; use a sample application purge of ~2-3 inches Hg.

**Sample Wash**

1. Pipette 250 µL D.I. water; purge using ~4-6 inches Hg.
2. Purge to complete dryness using ~10 inches Hg for ~10 min.

**Sample Elution**

1. Insert collection tube insert tray and collect eluent using 200 µL glass auto sampler micro vial inserts.
2. Pipette 150 µL MeOH to elute the sample; purge slowly using ~2-3 inches Hg.

**Solvent Evaporation and Sample Reconstitution**

1. DNA Speed Vac^®^ micro vial insert eluent to complete dryness (~20 min at 65°C).

2. Reconstitute with 25 µL MethElute™ solution.

3. Place microvial insert into standard autosampler vial.

4. Cap and vortex ~15 sec; inject 1 µL into GC-MS/MS system for dA analysis.

**Set-points for Agilent 7890A GC, 7693 Autosampler and 7000B MS Triple Quad for dA Analysis**

**GC inlet**

Mode – pulsed split-less

Temperature – 235°C

Injection pulse pressure – 25 psi until 1 min

Purge flow to split vent – 50 mL/min helium at 1.1 min

Total flow – 54 mL/min helium

Septum purge flow – 2-3 mL/min helium

Gas saver – 15 mL/min helium after 2 min

Aux 1 Headspace G3520 inlet – N/A

**GC program for DNA (dA)**

Initial GC oven temp – 50°C

Initial oven hold time – 1 min

Rate – 170°C/min

Final GC oven temp – 320°C

Final GC oven hold time – 1.5 min

Run time – 4.5 min

Equilibration time – 0.1 min

GC transfer line - 320°C

Oven max temperature – based on the column phase

**GC column used for DNA (dA)**

LTM Column – DB-17ms, 15 m x 0.25mm I.D. x 0.25 µm film

Head pressure – 16.5 psi

Flow – 1 mL/min helium (constant)

Average velocity – 49.97 cm/sec

Holdup time – 0.5 min

**GC autosampler**

Syringe size – 10 µL

Injection volume – 1 µL

Post washes A – 3 with ethanol

Post washes B – 3 with acetone

Sample washes – 1

Sample wash volume - 2 µL

Sample pumps – 3

Viscosity delay – 3 sec

Air gap – 0.2 µL

**GC autosampler barcode mixer**

Mixer – enabled

Mixer cycle – 1

Mixer time – 10 sec

Mixer speed – 2,000 rpm

**MS set-points**

Helium quench gas – 2.25 mL/min

Nitrogen collision gas – 1.5 mL/min

Isobutane reagent gas – 40% or 2 mL/min

Ion source temp – 350°C

Quad 1 temp – 150°C

Quad 2 temp – 150°C

Ion source – chemical ionization (CI)

Mode – positive ion (CI+)

Electron energy mode – use tune setting

Emission current – 240 µA

Solvent delay – 2.5 min

Run time – 4.5 min

Time filter enabled – none

Peak Width – 0.7

MS 1 resolution – unit

MS 2 resolution – unit

Dwell time – 20 msec

Scan rate – 9.5 cycles/sec

Collision energy – 15 V

Gain – none

Electron multiplier (V) – use tune setting

Delta EMV (V) – +400 above tune setting

HED - -10 kV

Rough vacuum – 1.86E+2 mTorr

High vacuum – 8.46E-5 Torr

**Instrument Tuning (Mass Calibration)**

Calibrate using PCI autotune, PFDTD calibrant, methane reagent gas

Tune file – isobutane.pci.tune.xml

Tune MS weekly or as necessary (e.g. source cleaning)

**Full Scan Mode**

Mass scan range – 50 to 400 m/z

**Multiple Reaction Monitoring (MRM) transitions**

dA (M0) derivative – precursor and product ion (m/z 308/164)

dA (M+1) derivative – precursor and product ion (m/z 309/164)

dA (M+2) derivative – precursor and product ion (m/z 310/164)

dA (M+5) derivative – precursor and product ion (m/z 313/169)

**Set-points for Agilent 7697A Headspace Sampler**

**Temperature**

Oven – 80°C

Loop/Valve - 105°C

Transfer line - 105°C

**Time**

GC cycle time – 5 min

Vial equilibrium time – 5 min

Pressure equilibrium time – 0.5 min

Inject time – 0.5 min

**Vial**

Fill mode – flow to pressure

Fill pressure – 15.0 psi

Fill flow – 50.0 mL/min

Loop fill mode – default

Loop fill ramp rate – 40.0 mL/min

Loop final pressure – 5.1 psi

Loop equilibrium – 0.05 min

Vent after extraction – No

Vial size – 10 mL

**GC Carrier**

Pressure – 4.8 psi at headspace oven 80°C and GC initial conditions with EPC control

**Other Parameters**

Loop volume – 1 mL

Extraction mode – Single

Purge flow – 100.0 mL/min

Purge time – 1.0 min

APG polarity – active high

Vial standby flow – 20.0 mL/min

**Set-points for Agilent 7890A GC and 7000B MS Triple Quad for Total Body Water (TBW) Analysis**

**GC inlet**

Inlet temperature – 150°C

Inlet pressure – 3.4 psi

Inlet liner – split liner 4 mm ID single gooseneck, deactivated glass wool, low pressure drop

Mode – split

Split ratio – 50:1

Split flow – 50 mL/min

Aux 1 Headspace G3520 inlet - 150°C

Total flow – 53 mL/min helium

Septum purge flow – 2 mL/min helium

Gas saver – 15 mL/min helium after 2 min

**GC oven program - isothermal**

LTM column temp - 80°C

GC oven temp – 80°C

GC oven hold time – 2.0 min

Run time – 2.0 min

Equilibration time – 0 min

GC oven max temperature - 340°C

**GC column**

Column – Agilent DB-17ms, 15 m x 0.25mm I.D. x 0.25 µm film

Flow rate – 1.0 mL/min helium (constant)

Average linear velocity – 48.2 cm/sec

Column holdup time – 0.5 min

Aux 2 MS transfer line – 150°C

**MS set-points**

Helium quench gas – 2.25 mL/min

Nitrogen collision gas – 1.5 mL/min

Isobutane reagent gas – 40% or 2 mL/min

MS source temp – 150°C

Quad 1 temp – 150°C

Quad 2 temp – 150°C

MS source – chemical ionization (CI)

Mode – negative ion (CI-)

Emission current – 50 µA

Electron energy mode – use tune setting

Solvent delay – 0 min

Run time – 2 min

Time filter enabled – yes

Peak width – 1 sec

Baseline subtraction - automatic

MS 1 resolution – unit

MS 2 resolution – unit

Full scan mode – 40 to 70 m/z

Scan time – 200 ms

Step size – 0.1 amu

Threshold - none

Profile data - yes

Scan rate – 5.0 cycles/sec

Gain – none

Electron multiplier – use tune setting

EMV – 1092 V

Delta EMV – +400 V

HED – 10 kV

Rough vacuum – 1.86E+2 mTorr

High vacuum – 8.46E-5 Torr

**MS Instrument Tuning (Mass Calibration)**

Calibrate using NCI autotune, PFDTD calibrant, and methane reagent gas

Tune file – isobutane.atunes.nci.tune.xml

Tuned MS weekly or as necessary (e.g. source cleaning)
